# Antipsychotic and pharmacogenomic effects on cross-sectional symptom severity and cognitive ability in schizophrenia

**DOI:** 10.1016/j.ebiom.2025.105745

**Published:** 2025-05-09

**Authors:** Siobhan K. Lock, Djenifer B. Kappel, Michael J. Owen, James T.R. Walters, Michael C. O'Donovan, Antonio F. Pardiñas, Sophie E. Legge

**Affiliations:** Centre for Neuropsychiatric Genetics and Genomics, Cardiff University, Hadyn Ellis Building, Maindy Road, Cardiff, Wales, CF24 4HQ, United Kingdom

**Keywords:** Schizophrenia, Pharmacogenomics, Antipsychotics, Clozapine, Symptom severity, Cognition

## Abstract

**Background:**

People with schizophrenia differ in the type and severity of symptoms experienced, as well as their response to medication. A better understanding of the factors that influence this heterogeneity is necessary for the development of individualised patient care. Here, we sought to investigate the relationships between phenotypic severity and both medication and pharmacogenomic variables in a cross-sectional sample of people with schizophrenia or schizoaffective disorder depressed type.

**Methods:**

Confirmatory factor analysis derived five dimensions relating to current symptom severity (positive symptoms, negative symptoms of diminished expressivity, negative symptoms of reduced motivation and pleasure, depression and suicide) and cognitive ability in participants prescribed with antipsychotic medication. Linear models were fit to test for associations between medication and pharmacogenomic variables with dimension scores in the full sample (N = 585), and in a sub-sample of participants prescribed clozapine (N = 215).

**Findings:**

Lower cognitive ability was associated with higher chlorpromazine-equivalent daily antipsychotic dose (β = −0.12; 95% CI, −0.19 to −0.05; *p* = 0.001) and with the prescription of clozapine (β = −0.498; 95% CI, −0.65 to −0.35; *p* = 3 × 10^−10^) and anticholinergic medication (β = −0.345; 95% CI, −0.55 to −0.14; *p* = 8 × 10^−4^). We also found associations between pharmacogenomics-inferred cytochrome P450 (CYP) enzyme activity and symptom dimensions. Increased genotype-predicted CYP2C19 and CYP3A5 activity were associated with reduced severity of the positive (β = −0.108; 95% CI, −0.19 to −0.03; *p* = 0.009) and both negative symptom dimensions (β = −0.113; 95% CI, −0.19 to −0.03; *p* = 0.007; β = −0.106; 95% CI, −0.19 to −0.02; *p* = 0.012), respectively. Faster predicted CYP1A2 activity was associated with higher cognitive dimension scores in people taking clozapine (β = 0.17; 95% CI, 0.05–0.29; *p* = 0.005).

**Interpretation:**

Our results confirm the importance of taking account of medication history (and particularly antipsychotic type and dose) in assessing potential correlates of cognitive impairment or poor functioning in patients with schizophrenia. We also highlight the potential for pharmacogenomic variation to be a useful tool to help guide drug prescription, although these findings require further validation.

**Funding:**

10.13039/501100000265Medical Research Council (MR/Y004094/1) and The National Center for Mental Health, funded by the 10.13039/100015846Welsh Government through Health and Care Research Wales. SKL was funded by a PhD studentship from 10.13039/100009981Mental Health Research UK (MHRUK). DBK, JTRW, MCOD and AFP were supported by the 10.13039/501100007601European Union’s Horizon 2020 research and innovation programme under grant agreement 964874.

**Research in context**

**Evidence before this study**

Pharmacogenomics has the potential to improve therapeutic outcomes in people diagnosed with schizophrenia, a disorder that displays great variation in phenotype severity and response to antipsychotics. Associations between certain cytochrome P450 (CYP) pharmacogenomic variables have already been demonstrated with overall schizophrenia severity in people with treatment-resistant schizophrenia. Past work focuses on patients taking clozapine. However, it is not known whether these associations are apparent across non-clozapine antipsychotics, if they are specific to different symptom domains, or whether any associations exist with cognitive ability in schizophrenia.

**Added value of this study**

This research explores medication and pharmacogenomic associations with schizophrenia phenotype dimensions in people being treated with antipsychotic medication. We examine these associations in a mixed-medication naturalistic sample, as well as a sub-group restricted to those currently prescribed clozapine. Our work benefits from a large sample size, both across the total sample and in the clozapine sub-group. Our dataset was deeply phenotyped, with rich information about symptom severity and cognitive ability. This allowed us to interrogate associations with different schizophrenia phenotypes, as opposed to a general measure of symptom severity.

**Implications of all the available evidence**

Our study provides preliminary evidence that routinely collected information (e.g., drug doses and medication prescription history) could be an important tool to help identify patients who may be at higher risk of cognitive impairment from schizophrenia pharmacotherapy. High-risk individuals could benefit from cognitive monitoring and mitigations to help offset this, including modifying prescriptions (e.g., avoiding prophylactic anticholinergic use, lowering antipsychotic dose, changing medication) or cognitive remediation therapy. Our results also provide preliminary support that antipsychotic prescription could be further guided by pharmacogenomic information, notably CYP1A2 activity scores in people prescribed with clozapine. Validation of both the medication and pharmacogenomic results in larger, more diverse datasets is a crucial next step before building a comprehensive picture of how these variables influence cognition and other schizophrenia phenotypes.

## Introduction

Schizophrenia is characterised by positive, negative, and disorganised symptoms alongside affective features and cognitive deficits. The presentation of schizophrenia is heterogeneous in the range and severity of symptoms and in the degree of response to antipsychotic treatment. There is evidence that genetic factors influence heterogeneity in treatment response, with one study suggesting that higher common variant liability to schizophrenia, as indexed by polygenic scores (PGS), is associated with poorer response to antipsychotics after 12 weeks of treatment.^1^ Similarly, both longitudinal and cross-sectional research has demonstrated an association between higher schizophrenia PGS and greater severity of negative and cognitive phenotype dimensions.^2^, ^3^, ^4^

**Fig. 1:**
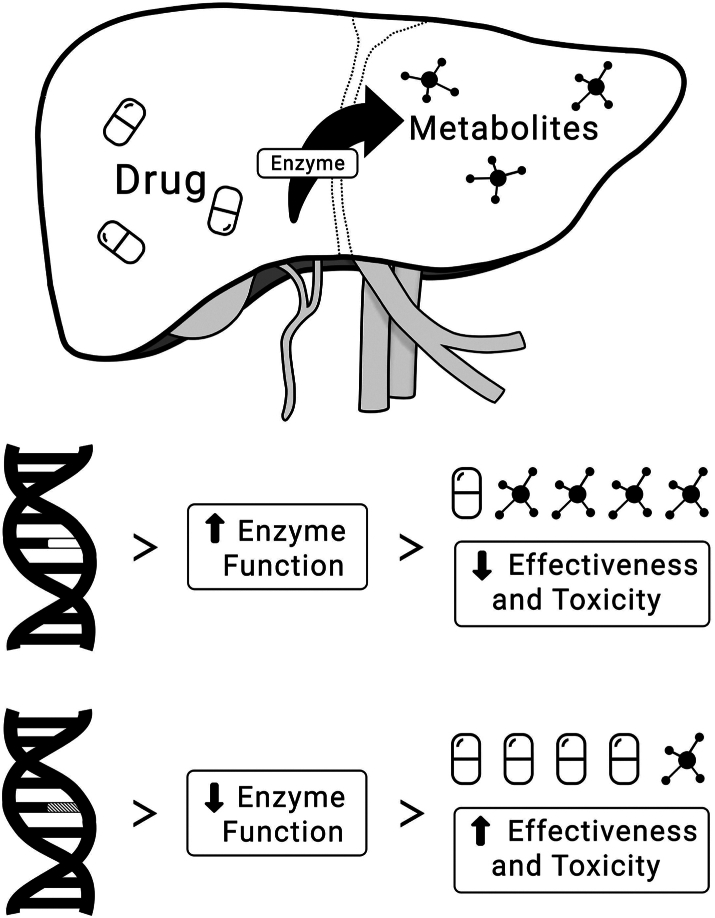
**Simplified diagram showing the impact of genetic variation on drug metabolism in the liver**. The majority of CYP-mediated drug metabolism occurs hepatically. Pharmacogenomic variation can result in increased, decreased, or no change to enzyme activity. Increased enzyme activity leads to faster metabolism, resulting in a lower drug concentration. This can be expected to lead to its reduced effectiveness but also a reduced risk for toxicity and adverse drug reactions. Conversely, variation leading to reduced enzyme activity results in slower metabolism of enzyme substrates. This can result in higher drug concentrations that might result in increased effectiveness but also increased risk of toxicity. The exact consequences of pharmacogenomic variation depend on the enzyme, drug, and metabolite(s) in question; for example, effects might vary if the metabolite has the potential to cause side effects or if the administered drug is a prodrug.

Beyond polygenic risk, variation in genes encoding proteins key to pharmacokinetic or pharmacodynamic processes (“pharmacogenes”) may also influence response to antipsychotic medication.^5^ Pharmacogenomic star alleles describe single or multiple genetic markers that alter the function of proteins influencing drug response. The most widely researched pharmacogenes are within the cytochrome P450 (CYP) family, which are implicated in drug metabolism and are highly variable within and across populations. Drug metabolism pathways are often well-studied biochemically and pharmacogenomic variation in relevant enzymes have been associated with antipsychotic pharmacokinetics.^6^, ^7^, ^8^ However, the extent to which between-person variability in these pathways influences drug effectiveness is still unclear. Fig. 1 illustrates how a pharmacogenomic alteration in a key metabolic enzyme might affect response to a medication. While such effects are intuitive and form the basis of successful prospective trials,^9^ as of April 2025, only 10 antipsychotics have regulator-approved guidelines with actionable pharmacogenomic markers in the PharmGKB database.^10^ Reviews from expert consortia report a similar number of drugs where pharmacogenomic information might be of use in clinical settings, though they also highlight the paucity of studies in the area.^11^

Establishing a pharmacogenomic marker as ‘actionable’ requires an evaluation of its effects on phenotypes beyond drug metabolism and pharmacokinetics, and studies on clinical outcomes can be valuable in these decisions.^11^ There is preliminary evidence that leveraging pharmacogenomic star alleles to infer enzyme activity may help predict schizophrenia symptom severity in those taking clozapine, the only evidence-based medication for treatment-resistant schizophrenia (TRS). Clozapine has a complex metabolic pathway but most of its first-pass bioconversion is driven by CYP1A2, with minor contributions of CYP2C19 and CYP2D6.^12^ Two recent studies reported associations between schizophrenia symptom severity and genotype-predicted enzyme activity for CYP1A2^13^ and CYP2C19^14^ in people with TRS taking this medication. However, the effects reported were inconsistent between studies, and their interpretation is complicated by differences in statistical methodologies and symptom rating scales used.

The present study aims to investigate possible links between medication and pharmacogenomic variables with current schizophrenia symptom severity and cognitive ability in a UK-based sample of individuals treated with antipsychotic medication. Although cross-sectional, the inclusion of pharmacogenomic information alongside medication variables offers the potential to inform the causal direction of associations. We expanded on existing literature by investigating associations in a mixed-medication group; however, we also reproduced previous pharmacogenomic analyses^13^^,^^14^ in a subsample restricted to patients prescribed clozapine. Our work follows recent calls to overcome “one size fits all” approaches in psychopharmacology by directly searching for predictors of clinical outcomes and pharmacodynamics,^15^^,^^16^ as this is essential to advancing the field of precision psychiatry through effective patient stratification.

## Methods

### Participants

**Fig. 2:**
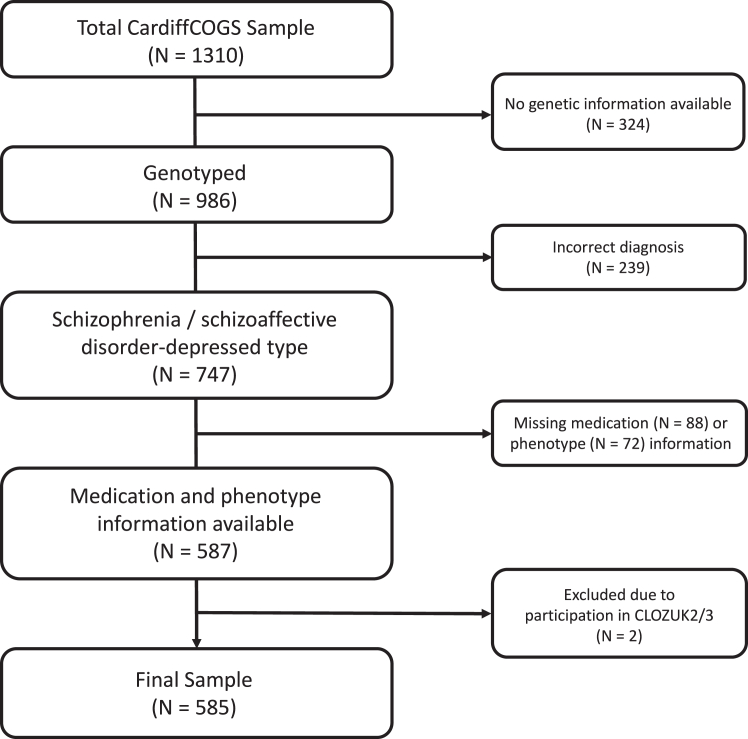
**Ascertainment of sample size**. Flow chart showing the ascertainment of the final sample, including sample size following each stage of inclusion/exclusions.

We included 585 individuals from the Cardiff COGnition in Schizophrenia (CardiffCOGS) cohort (Fig. 2). Participants were recruited from inpatient and community adult mental health services and voluntary services across the UK. Patients were also recruited from clozapine clinics; therefore, the cohort was enriched for those with treatment resistance (TRS; N = 215; 37%). All participants completed a clinical research interview based on the Schedules for Clinical Assessment in Neuropsychiatry^17^ (SCAN) and provided a blood sample for genetic analyses. All participants met DSM-IV^18^ or ICD-10^19^ diagnoses for schizophrenia or schizoaffective disorder, depressed type based on the SCAN-based interview and clinical case note review. A full description of the cohort with additional details is provided elsewhere.^3^ This study followed the STrengthening the REporting of Genetic Association Studies reporting recommendations^20^ (STREGA), an extension of the STROBE Statement.^21^ The checklist can be found in the Supplementary Materials.

### Phenotype data

The severity of positive, negative, affective, and disorganised symptoms was assessed at the time of the interview and are referred to hereafter as “current” symptoms. Symptoms were rated using the Scale for the Assessment of Positive Symptoms^22^ (SAPS), the Scale for the Assessment of Negative Symptoms^23^ (SANS), and the Calgary Depression Scale for Schizophrenia.^24^ Cognitive ability was assessed using the MATRICS Consensus Cognitive Battery^25^ (MCCB). Missing values for MATRICS variables were standardised against unaffected controls and then imputed in accordance with guidance from the MCCB handbook (see Supplementary Materials, M1). Symptom ratings and cognitive assessments were conducted by trained psychiatrists or psychology graduates and supervised by consultant psychiatrists, as described previously.^3^

All participants were treated with antipsychotics at the time of the interview. Medication data were obtained via self-report and case note review. A binary medication adherence variable was created from an ordinal questionnaire response that was based on the Medication Adherence Rating Scale,^26^ where adherent participants were defined as those who reported rarely missing doses, or who were medication supervised (see Supplementary Materials, M2). Antipsychotic doses were converted to chlorpromazine equivalents using the *ChlorpromazineR* package.^27^ Doses were converted based on international consensus values where available, and World Health Organisation Daily Defined Dose variables otherwise.^28^^,^^29^ Where individuals reported taking multiple antipsychotics concurrently, each antipsychotic dose was converted and then summed to give the total chlorpromazine-equivalent dose. Participants (N = 88; Fig. 2) were excluded when (i) no adherence information was available or when, for any antipsychotic reported: (ii) the drug name was not documented or (iii) no dose information was reported.

A binary index for the presence/absence of current anticholinergic drug use was created based on the current prescription of Procyclidine, Hyoscine, Benztropine, Benzhexol, or Pirenzepine. The Global Assessment Scale^30^ was used as a measure of current functioning. The UK census high-level ethnic group categories were used for ethnicity self-reports.

### Genetic data

Samples were extracted by Tepnel Pharmaceutical Services, KBiosciences UK/LGC Genomics, and in-house using either spin-column based DNA extraction (Tepnel Pharmaceutical Services and KBiosciences) or Nucleon BACC genomic DNA extraction kits (in-house). Participants were genotyped with the Illumina HumanOmniExpressExome v8 or the Illumina HumanOmniExpress v12 (Illumina Inc, USA) at the Broad Institute of Harvard and MIT, MA, USA and deCODE Genetics, Reykjavík, Iceland. This procedure and the curation and harmonisation of data from different arrays have been described previously.^31^ Combined genetic data for all samples were processed using the DRAGON-data quality-control pipeline “GenotypeQCtoHRC”^32^ with default parameters. Pre-imputation quality control was performed as follows; SNPs were excluded due to call rates <0.95, Minor Allele Frequency (MAF) < 0.01, or a Hardy–Weinberg Equilibrium mid-*p* < 10^−6^. Individuals with genotyping coverage rates <0.95 or potential errors in a PLINK “sex check” analysis (i.e., ambiguous genetic sex, or mismatch between self-reported and genetic sex) were excluded.

The Michigan Imputation server^33^ and Minimac-4 were utilised for imputation, using the Haplotype Reference Consortium v1.1 as the reference panel. Pre-imputation statistical phasing was carried out in the Michigan built-in service using Eagle 2.4.^33^ Post imputation, SNPs were removed when they had a hard-call genotype probability threshold <0.9, R^2^ < 0.9, MAF <0.01, and genotyping rates <0.95. Relatedness was assessed through the PC-Relate algorithm^34^ and a cutoff of π > 0.4 was used to remove related individuals.^3^ For pharmacogenomic allele calling we retained any monomorphic variants in the dataset and used a more relaxed imputation quality threshold (R^2^ < 0.7; Hard-call genotype probability <0.8). This allowed us to retain a greater number of pharmacogenomic informative SNPs to increase the allele calling accuracy, while still being stringent enough to avoid imputation errors in common genetic variants.^35^

PGS for schizophrenia,^36^ intelligence,^37^ and educational attainment^38^ were calculated using PRS-CS^39^ and PLINK v1.9.^40^ SNP effect sizes (BETA/Odds Ratio) were used alongside standard errors to compute posterior effect sizes in PRS-CS. The 1000 Genomes Project phase 3 European Linkage Disequilibrium (LD) reference panel was used to account for linkage disequilibrium.^41^ Additional parameters in PRS-CS included 10,000 burn-in iterations, 25,000 Markov chain Monte Carlo iterations, and a global shrinkage parameter phi of 1 for schizophrenia or “auto” for intelligence and educational attainment. Effect sizes generated in PRS-CS were passed to PLINK v1.9 for scoring. PGS were based on custom GWAS datasets from which all participants of the current study had been excluded.

To account for population stratification, a subset of common SNPs with high imputation quality (MAF >0.05, INFO >0.9), and low levels of LD (r^2^ < 0.2) was selected to calculate genetic principal components using the principal component analysis function in PLINK v2.^40^

### Pharmacogenomics

Pharmacogenomic markers for enzymes known to metabolise antipsychotic drugs (CYP1A2, CYP2C9, CYP2C19, CYP2D6, and CYP3A5) were called in PyPGx v0.20.0^42^ in Python v3.9.2.^43^ Allele calls made in PyPGx are based on information curated by the Clinical Pharmacogenomics Implementation Consortium (CPIC) and PharmGKB. The *run-chip-pipeline* command was used to screen genotype data for all pharmacogenomic markers which are not structural variants. Where available (e.g., CYP2D6, CYP2C9), activity scores were mapped to pharmacogenomic alleles called using the allele table within the PyPGx package. For the remaining enzymes, the mapping of pharmacogenomic alleles to activity scores was based on existing literature^13^^,^^44^ or inferred from PharmGKB Allele Functionality Reference Tables (see Supplementary Table S1). For *CYP1A2*, no guidance was available for ∗1K, therefore it was assigned the same activity score as ∗1C, also a decreased function allele. Where *CYP1A2*∗1F/1C was called on a single haplotype, we assigned an activity score of 1 based on past work.^45^

### Statistics

The sample size was maximal given missing phenotypic data, and in line with previous research using CardiffCOGS.^3^ Based on the smallest significant effect size detected in previous work^14^ assessing schizophrenia symptom severity and genotype-predicted enzyme activity (d = 0.25), our sample size is sufficient to achieve over 80% power based on a two-sample t-test. Data were analysed using R v4.4.0 in R Studio 2024.04.0 Build 735.^46^ Confirmatory factor analysis (CFA) was used to derive a latent factor model for current symptom severity in schizophrenia based on well-established and replicated latent models.^3^^,^^4^^,^^47^^,^^48^ Variables contributing to the model were global symptom measures from the SAPS and SANS, the self-report depressed mood and suicidal ideation and acts items from the Calgary Depression Scale for Schizophrenia, and the domain scores (excluding social cognition) from MATRICS. Ratings from SAPS, SANS, and Calgary Depression Scale for Schizophrenia were ordinal (0–5, SAPS and SANS; 0–3, Calgary Depression Scale for Schizophrenia). MATRICS variables were continuous. Patients with missing information across these phenotype variables were excluded from the analysis (N = 72; Fig. 2). *lavaan* v0.6.17 was used to fit the CFA, using default settings.^49^ Dimension scores based on the best fit CFA model were calculated for each participant. Model fit was guided by the Comparative Fit Index, Root Mean Square Error of Approximation, and the Standardised Root Mean Squared Residual. We would expect that current phenotype severity, indexed by latent variable scores, would be associated with functional impairments. Therefore, as an in-sample validity check of our derived variables, we regressed each factor against the Global Assessment Scale.

Linear regression models were used to test for associations between medication variables and symptom dimensions. Medication variables included daily chlorpromazine-equivalent antipsychotic dose, clozapine use, and anticholinergic use. All continuous variables were standardised, and all models controlled for schizophrenia PGS, medication adherence, age, sex (self-reported), and the first 5 genetic principal components.^50^ Finally, intelligence and educational attainment PGS were used as an estimate for premorbid intelligence in the analyses investigating the cognitive ability dimension. We chose these PGS as together they are the strongest genetic predictor of intelligence in previous research,^51^^,^^52^ and neither variable was associated with chlorpromazine-equivalent antipsychotic dose in our total sample. We also considered using premorbid intelligence estimated from the National Adult Reading Test (NART)^53^ via the recommended transformation (IQ = 130.6-1.24∗NART error score). This measure was associated with dose indicating the possibility that antipsychotic dose influenced performance on the NART or vice versa. Nevertheless, we provide an alternative analysis using premorbid intelligence as estimated by the NART. In addition, we performed a sensitivity analysis to assess whether associations between medication variables and phenotype severity were influenced by covarying for a binary diagnosis variable (schizoaffective disorder depressed-type = 0, schizophrenia = 1).

The model was extended by including activity scores for CYP1A2, CYP2C9, CYP2C19, CYP2D6, and CYP3A5 in the total sample, and in the subgroup of participants prescribed clozapine (N = 215) separately. Note that analyses of the effects of the pharmacogenomic variables are adjusted for dose, clozapine use, and anticholinergic use. Finally, we performed a sensitivity analysis to test whether our pharmacogenomic models changed after controlling for concomitant medication or lifestyle factors that may influence enzyme function (phenoconversion^54^). We accounted for phenoconversion using two methods, (i) statistically through an interaction term between the enzyme activity score and a binary variable representing use of the relevant drug, and (ii) traditionally by replacing standard activity scores with phenoconversion-corrected activity scores. Phenoconversion-corrected activity scores were calculated based on work by Lesche and colleagues.^13^ Scores were multiplied by 1.5 in the presence of an inducer, and by 0 when a strong inhibitor was present. Models were fit only where a pharmacogenomic variable was associated with a phenotype dimension in our previous analyses.

### Ethics

CardiffCOGS received approval from the South-East Wales Research Ethics Committee (07/WSE03/110) and all participants provided written informed consent.

### Role of funders

The funders had no role in study design, data collection, data analyses, interpretation, or writing of this manuscript.

## Results

We included 585 participants with schizophrenia or schizoaffective disorder, depressed type (205 [35%] female; mean [SD] age of 43.5 [11.7] years). All participants were from the UK. Of the participants 98.3% reported being White, 0.2% reported being Black, Black British, Caribbean or African, 0.5% reported being Asian or Asian British, and 1% reported being Mixed or Multiple ethnic groups.

At the time of the interview, participants were prescribed at least one of 16 different antipsychotics, administered orally or by long-acting injection. Clozapine (N = 215), olanzapine (N = 103), and aripiprazole (N = 74) were the most common antipsychotics reported. Furthermore, some patients were prescribed multiple antipsychotics concurrently (N = 99); of these, the most common drug combinations were clozapine augmented with either amisulpride (N = 22) or aripiprazole (N = 20). Demographic and descriptive information is displayed in Table 1, with the frequency of antipsychotics reported and the frequency of pharmacogenomic star alleles in Supplementary Tables S2 and S3, respectively.

**Table 1:** Demographics and descriptive statistics for key variables.

Variable	Overall N = 585	Males N = 380	Females N = 205
Age (years)	43.5 (11.7)	43.4 (11.8)	43.8 (11.7)
Chlorpromazine-Equivalent Antipsychotic Dose (mg/day)	587.6 (390.1)	599.1 (401.0)	566.3 (369.2)
Diagnosis			
Schizophrenia	485 (83%)	338 (89%)	58 (28%)
Schizoaffective disorder depressed-type	100 (17%)	42 (11%)	147 (72%)
Clozapine use			
No	370 (63%)	237 (62%)	133 (65%)
Yes	215 (37%)	143 (38%)	72 (35%)
Anticholinergic use			
No	494 (85%)	329 (87%)	165 (80%)
Yes	91 (16%)	51 (13%)	40 (20%)
Adherent			
No	31 (5.3%)	24 (6.3%)	7 (3.4%)
Yes	554 (94.7%)	356 (94%)	198 (97%)
Antipsychotic polypharmacy			
No	486 (83%)	322 (85%)	164 (80%)
Yes	99 (17%)	58 (15%)	41 (20%)
Cigarette use			
No	235 (40%)	141 (37%)	94 (46%)
Yes	305 (52%)	205 (54%)	100 (49%)
Unknown	45 (8%)	34 (9%)	11 (5%)
Fluoxetine use			
No	551 (94%)	363 (96%)	188 (92%)
Yes	34 (6%)	17 (4%)	17 (8%)
Carbamazepine use			
No	580 (99%)	377 (99%)	203 (99%)
Yes	5 (1%)	3 (1%)	2 (1%)

Demographic variables and descriptive statistics for key variables in the total sample (N = 585) and split by self-reported sex. Mean (SD) is reported for continuous variables and n (%) is reported for categorical variables.

### Confirmatory factor analysis of schizophrenia phenotypes

**Fig. 3:**
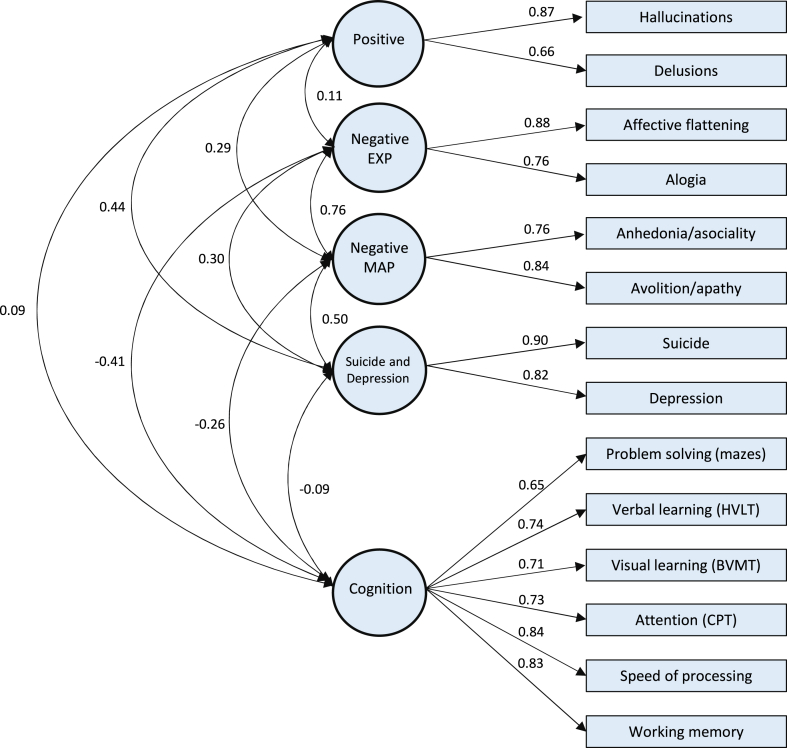
**Factor structure from the confirmatory factor analysis of schizophrenia phenotypes**. Factor structure from the confirmatory factor analysis of current schizophrenia symptom severity and cognitive ability within the CardiffCOGS sample (N = 585). Standardised loadings between latent factors (circles) and contributing phenotypes (rectangles) are reported on straight lines. Standardised factor loadings represent regression coefficients in a latent variable model and range from −1 to +1, with values closer to 1 indicating a stronger positive relationship; all loadings in the model are significant at *p* < 0.001. Curved lines represent correlations between latent factors. Values range between 0 and 1, with values closer to 1 indicating a stronger relationship between the latent factors. All correlations were significant at *p* < 0.001, except for that between the positive and expressivity dimensions (*p* = 0.073), the positive and cognitive dimensions (*p* = 0.113), and the cognitive and suicide and depression dimensions (*p* = 0.143). EXP, Diminished Expressivity; MAP, Reduced Motivation and Pleasure; HVLT, Hopkins Visual Learning Test; BVMT, Brief Visuospatial Memory Test-Revised; CPT, Continuous Performance Test.

Four factor structures were fit to determine which best represented the data. Descriptive statistics for the raw variables (i.e., symptom and phenotype items) going into the models, and factor loadings for each raw variable on the derived latent factors are found in Supplementary Table S4. The model with the best fit (Comparative Fit Index = 0.997, Root Mean Square Error of Approximation = 0.019, Standardised Root Mean Square Residual = 0.043) had five dimensions related to positive symptoms, negative symptoms of diminished expressivity, negative symptoms of reduced motivation and pleasure, depression and suicide, and cognitive ability (Fig. 3); we have defined phenotype severity as the domain scores derived from this best-fit confirmatory factor analysis. The derived factors were all significantly associated with the Global Assessment Scale in the expected direction (Supplementary Materials, R1).

### Associations between phenotype dimensions and medication variables

All results below are from linear regression models and are reported as standardised effect sizes (β) and uncorrected *p* values. Results tables contain both uncorrected- and FDR-corrected *p* values. Only associations that passed the 5% False Discovery Rate (FDR) threshold were considered significant.

The results of association tests between phenotype dimensions and medication variables are shown in Table 2. Higher scores on the positive dimension were associated with higher chlorpromazine-equivalent antipsychotic dose (β = 0.145; 95% CI, 0.06–0.23; *p* = 8 × 10^−4^). Higher scores on the diminished expressivity dimension were associated with clozapine use (β = 0.417; 95% CI, 0.25–0.59; *p* = 2 × 10^−6^). Higher scores on the reduced motivation and pleasure dimension were also associated with clozapine use (β = 0.232; 95% CI, 0.06–0.41; *p* = 0.009). Higher scores on the suicide and depression dimension were associated with higher chlorpromazine-equivalent antipsychotic dose (β = 0.099; 95% CI, 0.02–0.18; *p* = 0.021).

**Table 2:** Associations between medication variables and severity of the five examined schizophrenia phenotype dimensions.

Predictors	Positive		Diminished expressivity		Reduced motivation & pleasure		Depression & suicide		Cognition	
	β (SE)	*p* (FDR)	β (SE)	*p* (FDR)	β (SE)	*p* (FDR)	β (SE)	*p* (FDR)	β (SE)	*p* (FDR)
CPZ-eq antipsychotic dose (mg/day)	0.145 (0.04)	8 × 10^−4^ (0.004)	0.043 (0.04)	0.307 (0.307)	0.056 (0.04)	0.195 (0.244)	0.099 (0.04)	0.021 (0.034)	−0.12 (0.04)	0.001 (0.004)
Clozapine (Yes)	−0.061 (0.09)	0.491 (0.613)	0.417 (0.09)	2 × 10^−6^ (6 × 10^−6^)	0.232 (0.09)	0.009 (0.015)	−0.007 (0.09)	0.937 (0.937)	−0.498 (0.08)	3 × 10^−10^ (1 × 10^−9^)
Anticholinergic (Yes)	0.081 (0.12)	0.493 (0.493)	0.222 (0.12)	0.055 (0.076)	0.272 (0.12)	0.021 (0.053)	0.221 (0.12)	0.06 (0.076)	−0.345 (0.1)	8 × 10^−4^ (0.004)
Adherent (Yes)	−0.498 (0.19)	0.008 (0.038)	−0.235 (0.18)	0.201 (0.201)	−0.364 (0.19)	0.051 (0.107)	−0.345 (0.19)	0.064 (0.107)	0.26 (0.16)	0.11 (0.138)
Schizophrenia PGS	−0.041 (0.04)	0.335 (0.558)	0.029 (0.04)	0.495 (0.618)	−0.006 (0.04)	0.879 (0.879)	−0.063 (0.04)	0.138 (0.345)	−0.1 (0.04)	0.009 (0.043)
Intelligence PGS									0.075 (0.04)	0.091 (0.091)
Educational attainment PGS									0.13 (0.04)	0.003 (0.003)
Age	−0.048 (0.04)	0.261 (0.435)	0.061 (0.04)	0.147 (0.367)	0.036 (0.04)	0.395 (0.493)	0.001 (0.04)	0.985 (0.985)	−0.376 (0.04)	2 × 10^−22^ (1 × 10^−21^)
Sex (Female)	0.052 (0.09)	0.547 (0.684)	−0.176 (0.09)	0.04 (0.146)	−0.104 (0.09)	0.232 (0.386)	0.165 (0.09)	0.058 (0.146)	0.021 (0.08)	0.783 (0.783)
PC1	−0.026 (0.04)	0.534 (0.668)	0.009 (0.04)	0.835 (0.835)	−0.028 (0.04)	0.501 (0.668)	−0.065 (0.04)	0.126 (0.631)	0.029 (0.04)	0.429 (0.668)
PC2	−0.034 (0.04)	0.411 (0.594)	0.026 (0.04)	0.52 (0.594)	0.022 (0.04)	0.594 (0.594)	−0.054 (0.04)	0.192 (0.481)	−0.047 (0.04)	0.191 (0.481)
PC3	−0.062 (0.04)	0.132 (0.658)	0.008 (0.04)	0.835 (0.97)	0.008 (0.04)	0.85 (0.97)	0.002 (0.04)	0.97 (0.97)	−0.018 (0.04)	0.617 (0.97)
PC4	0.003 (0.04)	0.942 (0.942)	−0.031 (0.04)	0.455 (0.892)	−0.015 (0.04)	0.713 (0.892)	−0.021 (0.04)	0.616 (0.892)	0.041 (0.04)	0.264 (0.892)
PC5	−0.032 (0.04)	0.441 (0.551)	0.064 (0.04)	0.115 (0.315)	0.063 (0.04)	0.126 (0.315)	0.034 (0.04)	0.402 (0.551)	0.021 (0.04)	0.562 (0.562)
R2	0.045		0.068		0.042		0.041		0.274	
Adjusted R2	0.025		0.049		0.022		0.021		0.256	
N	585		585		585		585		585	

Associations between medication variables with schizophrenia symptom and cognitive ability dimensions in CardiffCOGS. Five separate models were fit, each with severity scores for a schizophrenia phenotype as the outcome variable. Variables of interest included the medication variables, chlorpromazine-equivalent daily antipsychotic dose, clozapine use, and anticholinergic use. All models controlled for medication adherence, schizophrenia PGS, age, sex, and the first five genetic principal components. Intelligence PGS and Educational Attainment PGS were included as additional covariates in the model where the cognitive ability dimension is the outcome. Standardised regression estimates are reported. CPZ-eq, chlorpromazine-equivalent; SE, Standard Error; PGS, Polygenic Score; FDR, False Discovery Rate.

Lower scores on the cognition dimension, indicating poorer cognitive ability, were associated with higher chlorpromazine-equivalent daily antipsychotic dose (β = −0.12; 95% CI, −0.19 to −0.05; *p* = 0.001), clozapine use (β = −0.498; 95% CI, −0.65 to −0.35; *p* = 3 × 10^−10^), and anticholinergic use (β = −0.345; 95% CI, −0.55 to −0.14; *p* = 8 × 10^−4^). These associations were not affected by using NART IQ to control for premorbid cognition in place of genetic predictors of intelligence (Supplementary Materials, R2).

A sensitivity analysis (Supplementary Table S5) demonstrated that statistically controlling for type of diagnosis (i.e., schizophrenia vs. schizoaffective disorder depressed-type) did not affect the associations between medication variables and phenotype severity. Schizophrenia diagnosis itself compared to schizoaffective disorder depressed-type was associated with lower scores on the suicide and depression dimension, but not with any of the other phenotype dimensions.

### Pharmacogenomic associations with the phenotype dimensions

All pharmacogenomic associations were adjusted for medication variables. The addition of pharmacogenomic variables into the models did not substantially influence the associations between the medication variables and schizophrenia phenotype dimensions described above. While some attenuation of *p* values was observed, there was no change to the overall pattern of results, with previously significant associations remaining so.

In the full sample, we found several associations in which lower symptom dimension scores, and so less severe symptoms, were associated with faster genotype-inferred enzyme activity. Lower scores on the positive symptom dimension were associated with higher CYP2C19 activity scores (β = −0.108; 95% CI, −0.19 to −0.03; *p* = 0.009). Lower scores on the diminished expressivity dimension were associated with higher CYP3A5 activity score (β = −0.113; 95% CI, −0.19 to −0.03; *p* = 0.007). Similarly, we also found an association between lower scores on the reduced motivation and pleasure dimension and higher CYP3A5 activity score (β = −0.106; 95% CI, −0.19 to −0.02; *p* = 0.012). There were no pharmacogenomic associations with either the suicide and depression or the cognitive ability dimension (Table 3).

**Table 3:** Associations between medication and pharmacogenomic variables and severity of the five examined schizophrenia phenotype dimensions.

Predictors	Positive		Diminished expressivity		Reduced motivation & pleasure		Depression & suicide		Cognition	
	β (SE)	*p* (FDR)	β (SE)	*p* (FDR)	β (SE)	*p* (FDR)	β (SE)	*p* (FDR)	β (SE)	*p* (FDR)
CPZ-eq Antipsychotic dose (mg/day)	0.146 (0.04)	7 × 10^−4^ (0.003)	0.035 (0.04)	0.405 (0.405)	0.045 (0.04)	0.296 (0.37)	0.098 (0.04)	0.023 (0.039)	−0.115 (0.04)	0.003 (0.006)
Clozapine (Yes)	−0.044 (0.09)	0.623 (0.778)	0.414 (0.09)	3 × 10^−6^ (8 × 10^−6^)	0.236 (0.09)	0.008 (0.014)	0.004 (0.09)	0.961 (0.961)	−0.499 (0.08)	4 × 10^−10^ (2 × 10^−9^)
Anticholinergic (Yes)	0.059 (0.12)	0.618 (0.618)	0.209 (0.12)	0.073 (0.104)	0.266 (0.12)	0.025 (0.062)	0.205 (0.12)	0.083 (0.104)	−0.329 (0.1)	0.002 (0.008)
CYP1A2 activity score	−0.051 (0.04)	0.227 (0.365)	−0.066 (0.04)	0.116 (0.289)	−0.039 (0.04)	0.365 (0.365)	−0.042 (0.04)	0.321 (0.365)	0.07 (0.04)	0.06 (0.289)
CYP2D6 activity score	−0.022 (0.04)	0.595 (0.744)	0.047 (0.04)	0.25 (0.67)	0.046 (0.04)	0.268 (0.67)	−0.008 (0.04)	0.847 (0.847)	−0.02 (0.04)	0.587 (0.744)
CYP3A5 activity score	−0.042 (0.04)	0.319 (0.333)	−0.113 (0.04)	0.007 (0.031)	−0.106 (0.04)	0.012 (0.031)	−0.041 (0.04)	0.333 (0.333)	0.037 (0.04)	0.321 (0.333)
CYP2C19 activity score	−0.108 (0.04)	0.009 (0.047)	−0.003 (0.04)	0.936 (0.936)	−0.04 (0.04)	0.339 (0.565)	−0.079 (0.04)	0.057 (0.143)	0.008 (0.04)	0.817 (0.936)
CYP2C9 activity score	−0.049 (0.04)	0.237 (0.54)	−0.012 (0.04)	0.764 (0.919)	0.004 (0.04)	0.919 (0.919)	−0.041 (0.04)	0.324 (0.54)	−0.048 (0.04)	0.188 (0.54)
Adherent (Yes)	−0.418 (0.19)	0.026 (0.122)	−0.257 (0.19)	0.167 (0.167)	−0.371 (0.19)	0.051 (0.122)	−0.34 (0.19)	0.073 (0.122)	0.257 (0.17)	0.121 (0.152)
Schizophrenia PGS	−0.044 (0.04)	0.3 (0.501)	0.021 (0.04)	0.615 (0.73)	−0.015 (0.04)	0.73 (0.73)	−0.063 (0.04)	0.144 (0.36)	−0.092 (0.04)	0.017 (0.083)
Intelligence PGS									0.084 (0.04)	0.06 (0.06)
Educational attainment PGS									0.123 (0.04)	0.006 (0.006)
Age	−0.052 (0.04)	0.218 (0.363)	0.059 (0.04)	0.162 (0.363)	0.033 (0.04)	0.444 (0.555)	−0.005 (0.04)	0.91 (0.91)	−0.375 (0.04)	9 × 10^−22^ (4 × 10^−21^)
Sex (Female)	0.053 (0.09)	0.541 (0.676)	−0.197 (0.09)	0.023 (0.115)	−0.126 (0.09)	0.151 (0.252)	0.147 (0.09)	0.094 (0.236)	0.03 (0.08)	0.698 (0.698)
PC1	−0.019 (0.04)	0.651 (0.724)	0.02 (0.04)	0.628 (0.724)	−0.015 (0.04)	0.724 (0.724)	−0.057 (0.04)	0.18 (0.724)	0.029 (0.04)	0.433 (0.724)
PC2	−0.032 (0.04)	0.442 (0.665)	0.022 (0.04)	0.595 (0.665)	0.018 (0.04)	0.665 (0.665)	−0.052 (0.04)	0.209 (0.539)	−0.045 (0.04)	0.216 (0.539)
PC3	−0.048 (0.04)	0.248 (0.728)	0.022 (0.04)	0.589 (0.728)	0.023 (0.04)	0.578 (0.728)	0.014 (0.04)	0.728 (0.728)	−0.018 (0.04)	0.615 (0.728)
PC4	0.007 (0.04)	0.866 (0.961)	−0.016 (0.04)	0.703 (0.961)	−0.002 (0.04)	0.961 (0.961)	−0.018 (0.04)	0.676 (0.961)	0.036 (0.04)	0.33 (0.961)
PC5	−0.03 (0.04)	0.466 (0.582)	0.076 (0.04)	0.064 (0.24)	0.069 (0.04)	0.096 (0.24)	0.036 (0.04)	0.388 (0.582)	0.012 (0.04)	0.751 (0.751)
R2	0.060		0.084		0.056		0.051		0.280	
Adjusted R2	0.032		0.056		0.027		0.022		0.256	
N	578		578		578		578		578	

Associations between medication and pharmacogenomic variables with schizophrenia symptom and cognitive ability dimensions in CardiffCOGS. Five separate models were fit, each with severity scores for a schizophrenia phenotype as the outcome variable. Variables of interest included the medication variables (i.e., chlorpromazine-equivalent daily antipsychotic dose, clozapine use, and anticholinergic use) and pharmacogenomic variables (i.e., the genetics-inferred enzyme activity scores). All models controlled for medication adherence, schizophrenia PGS, age, sex, and the first five genetic principal components. Intelligence PGS and Educational Attainment PGS were included as additional covariates in the model where the cognitive ability dimension is the outcome. Standardised regression estimates are reported. CPZ-eq, chlorpromazine-equivalent; SE, Standard Error; PGS, Polygenic Score; FDR, False Discovery Rate.

Within the subgroup of participants taking clozapine, higher CYP1A2 activity score was associated with higher cognitive ability (β = 0.17; 95% CI, 0.05–0.29; *p* = 0.005). No other pharmacogenomic variables were significantly associated with schizophrenia phenotype severity after correcting for multiple comparisons in this clozapine taking group (Table 4), although higher antipsychotic dose was nominally associated with lower cognitive ability (β = −0.147; 95% CI, −0.27 to −0.02; *p* = 0.02).

**Table 4:** Associations between medication and pharmacogenomic variables and severity of the five examined schizophrenia phenotype dimensions in the subgroup of participants prescribed clozapine.

Predictors	Positive (clozapine subgroup)		Diminished expressivity (clozapine subgroup)		Reduced motivation & pleasure (clozapine subgroup)		Depression & suicide (clozapine subgroup)		Cognition (clozapine subgroup)	
	β (SE)	*p* (FDR)	β (SE)	*p* (FDR)	β (SE)	*p* (FDR)	β (SE)	*p* (FDR)	β (SE)	*p* (FDR)
CPZ-eq Antipsychotic Dose (mg/day)	0.02 (0.07)	0.786 (0.786)	0.077 (0.07)	0.273 (0.683)	0.03 (0.07)	0.673 (0.786)	0.05 (0.07)	0.471 (0.785)	−0.147 (0.06)	0.02 (0.1)
Anticholinergic (Yes)	−0.076 (0.2)	0.704 (0.88)	0.023 (0.19)	0.907 (0.907)	0.137 (0.2)	0.486 (0.81)	0.189 (0.19)	0.327 (0.81)	−0.274 (0.17)	0.109 (0.547)
CYP1A2 activity score	−0.083 (0.07)	0.242 (0.404)	−0.108 (0.07)	0.117 (0.293)	−0.041 (0.07)	0.556 (0.556)	−0.059 (0.07)	0.387 (0.484)	0.17 (0.06)	0.005 (0.026)
CYP2D6 activity score	−0.028 (0.07)	0.695 (0.836)	0.071 (0.07)	0.315 (0.836)	0.064 (0.07)	0.369 (0.836)	−0.039 (0.07)	0.574 (0.836)	−0.013 (0.06)	0.836 (0.836)
CYP3A5 activity score	−0.082 (0.07)	0.251 (0.251)	−0.12 (0.07)	0.085 (0.198)	−0.103 (0.07)	0.147 (0.198)	−0.126 (0.07)	0.07 (0.198)	0.087 (0.06)	0.158 (0.198)
CYP2C19 activity score	−0.05 (0.07)	0.481 (0.843)	0.014 (0.07)	0.843 (0.843)	0.014 (0.07)	0.84 (0.843)	0.052 (0.07)	0.444 (0.843)	0.023 (0.06)	0.699 (0.843)
CYP2C9 activity score	−0.041 (0.07)	0.564 (0.62)	−0.164 (0.07)	0.019 (0.095)	−0.121 (0.07)	0.086 (0.215)	−0.098 (0.07)	0.155 (0.258)	0.03 (0.06)	0.62 (0.62)
Adherent (Yes)	−0.168 (0.52)	0.749 (0.749)	−0.306 (0.51)	0.549 (0.749)	−0.411 (0.52)	0.429 (0.749)	−0.36 (0.51)	0.479 (0.749)	0.226 (0.45)	0.615 (0.749)
Schizophrenia PGS	−0.021 (0.07)	0.772 (0.772)	0.022 (0.07)	0.748 (0.772)	−0.034 (0.07)	0.632 (0.772)	−0.135 (0.07)	0.053 (0.229)	−0.108 (0.06)	0.092 (0.229)
Intelligence PGS									0.14 (0.08)	0.076 (0.076)
Educational attainment PGS									0.083 (0.08)	0.279 (0.279)
Age	−0.135 (0.07)	0.06 (0.15)	0.057 (0.07)	0.41 (0.683)	0.017 (0.07)	0.813 (0.992)	0.001 (0.07)	0.992 (0.992)	−0.398 (0.06)	6 × 10^−10^ (3 × 10^−9^)
Sex (Female)	0.236 (0.15)	0.112 (0.279)	−0.192 (0.14)	0.182 (0.304)	−0.067 (0.15)	0.649 (0.812)	0.245 (0.14)	0.089 (0.279)	0.008 (0.13)	0.948 (0.948)
PC1	−0.036 (0.08)	0.631 (0.631)	−0.11 (0.07)	0.131 (0.164)	−0.114 (0.07)	0.127 (0.164)	−0.165 (0.07)	0.024 (0.121)	0.102 (0.06)	0.111 (0.164)
PC2	−0.087 (0.15)	0.566 (0.566)	−0.197 (0.15)	0.181 (0.302)	−0.232 (0.15)	0.123 (0.302)	−0.316 (0.15)	0.032 (0.16)	0.132 (0.13)	0.305 (0.381)
PC3	0.018 (0.29)	0.951 (0.951)	0.377 (0.28)	0.181 (0.302)	0.331 (0.29)	0.247 (0.309)	0.46 (0.28)	0.102 (0.254)	−0.41 (0.25)	0.097 (0.254)
PC4	−0.024 (0.28)	0.932 (0.932)	−0.448 (0.27)	0.1 (0.126)	−0.469 (0.28)	0.091 (0.126)	−0.467 (0.27)	0.086 (0.126)	0.396 (0.24)	0.097 (0.126)
PC5	0.053 (0.1)	0.61 (0.763)	−0.136 (0.1)	0.178 (0.444)	−0.216 (0.1)	0.036 (0.182)	0.052 (0.1)	0.601 (0.763)	0.019 (0.09)	0.833 (0.833)
R2	0.063		0.114		0.082		0.122		0.335	
Adjusted R2	−0.013		0.043		0.008		0.051		0.273	
N	215		215		215		215		215	

Associations between medication and pharmacogenomic variables with schizophrenia symptom and cognitive ability dimensions in the subgroup of CardiffCOGS participants prescribed clozapine. Five separate models were fit, each with severity scores for a schizophrenia phenotype as the outcome variable. Variables of interest included the medication variables (i.e., chlorpromazine-equivalent daily antipsychotic dose, clozapine use, and anticholinergic use) and pharmacogenomic variables (i.e., the genetics-inferred enzyme activity scores). All models controlled for medication adherence, schizophrenia PGS, age, sex, and the first five genetic principal components. Intelligence PGS and Educational Attainment PGS were included as additional covariates in the model where the cognitive ability dimension is the outcome. Standardised regression estimates are reported. CPZ-eq, chlorpromazine-equivalent; SE, Standard Error; PGS, Polygenic Score; FDR, False Discovery Rate.

We also provide unadjusted estimates for associations between pharmacogenomic variables and symptom dimensions (Supplementary Tables S6 and S7). Sensitivity analyses in which we controlled for medication that may lead to phenoconversion did not lead to different results (Supplementary Tables S8 and S9). However, inclusion of an interaction term between CYP1A2 and smoking status in our model attenuated the association between CYP1A2 activity score and the cognitive dimension (Supplementary Table S10). This suggests that this pharmacogenomic association may be partly explained by patient lifestyle factors. However, our sensitivity analysis is limited by incomplete smoking information in the sample; thus, diminished statistical power may also account for this weakened association (Supplementary Materials, R3).

## Discussion

This cross-sectional, exploratory study investigated medication and pharmacogenomic correlates of current phenotype severity in people with schizophrenia or schizoaffective disorder depressed-type. We found that higher daily chlorpromazine-equivalent antipsychotic dose was associated with more severe scores on both the positive symptom and the suicide and depression dimensions, while clozapine use was associated with worse scores on the two negative symptom dimensions (i.e., diminished expressivity, reduced motivation and pleasure). We also found that chlorpromazine-equivalent daily antipsychotic dose, clozapine use, and anticholinergic use were all associated with lower scores on the cognitive dimension, indicating poorer cognitive ability.

Some of the associations we have observed between drug dosage and symptomatology are likely to reflect influences of the latter on prescribing patterns. Higher doses of antipsychotic medication are likely to be prescribed to help manage more severe or incompletely resolved positive symptoms, while negative symptoms do not generally respond well to typical antipsychotics and may trigger the prescription of clozapine which does have efficacy in this domain.^55^

Our findings that antipsychotic dose, clozapine use, and anticholinergic use were associated with poorer cognition are consistent with other research.^56^, ^57^, ^58^, ^59^, ^60^, ^61^ Moreover, our effect sizes were, in some instances, substantial, with our strongest association indicating that scores on the cognitive ability dimension are nearly 0.5 standard deviations lower in people prescribed clozapine. If antipsychotic dose indeed influences cognitive ability, then reducing antipsychotic dose might benefit cognition if the risk of relapse can be balanced or mitigated.^62^ Indeed, both naturalistic research and randomised controlled trials have found evidence that antipsychotic dose reductions are associated with improved cognition in comparison to those on a maintained dose,^63^^,^^64^ and results from a recent meta-analysis are in line with these findings.^65^ This supports a causal interpretation of the inverse association between dose and cognitive ability.

However, interpreting cross-sectional findings relating to cognition and medication use is challenging due to the possibility of reverse causation whereby patients with cognitive impairment could be more likely to receive higher doses of medication. The situation with respect to clozapine may be even more complex because a prescription of clozapine usually necessitates a diagnosis of TRS, which is itself associated with greater cognitive impairment than treatment-responsive schizophrenia.^66^, ^67^, ^68^ Nevertheless, the associations observed between both antipsychotic dose and clozapine use with poorer cognition were significant after controlling for premorbid intelligence as indexed by PGS for intelligence and educational attainment. These medication associations mostly replicate in the alternative analysis described in the Supplementary Materials (R2) using NART to estimate premorbid intelligence, albeit with weaker signals. In all, our analyses tentatively support the interpretation that the association may be causally related to medication use, instead of reflecting the confounding effects of lower premorbid cognitive ability in those who subsequently receive clozapine or higher doses of antipsychotics.

Our combined analysis of pharmacogenomic and medication variables offers a potential means of drawing causal inferences regarding the observed associations between drug treatment and cognition. CYP1A2 is the main enzyme responsible for clozapine first-pass metabolism.^69^^,^^70^ Higher enzyme activity can be expected to result in lower clozapine bioavailability for a given dose, and a corresponding increase in its metabolites. Clozapine has a broad binding profile, antagonising, amongst others, muscarinic anticholinergic receptors.^71^ The M1 subtype is important to learning and memory, thus antagonism via clozapine suggests one route to cognitive impairment^72^; indeed, we observed a nominally significant association between higher cognitive ability and lower antipsychotic dose in those prescribed clozapine. In contrast, norclozapine, one of clozapine's major metabolites, activates some of the same muscarinic receptors that its parent drug blocks, particularly the M1 subtype.^73^^,^^74^ Under a causal model, a reduction in clozapine and an increase in norclozapine could be protective of cognitive ability,^75^^,^^76^ both being possible mechanisms for the observed association between higher cognitive ability and faster genotype-inferred CYP1A2 activity in people prescribed clozapine (Table 4). In the context of all these results, we remark that using pharmacogenetic information to phenotype enzyme activity, and thus drug metabolism, may highlight individuals that could be particularly vulnerable to dose-dependent adverse drug reactions (ADRs). We also note that while this research takes advantage of such a phenotyping approach, the atypical instances of clozapine metabolism that would characterise these individuals in clinical scenarios can already be detected by therapeutic drug monitoring.^77^ For this reason, we echo the recent recommendations for therapeutic drug monitoring procedures to be more readily adopted as a routine part of the clinical management of those on clozapine.^78^

We also reported an inverse association between prescription of anticholinergic medication and cognition. Some antipsychotics cause extrapyramidal side effects, generally as a dose-dependent ADR.^79^^,^^80^ Extrapyramidal side effects are managed with anticholinergic medication, and although not recommended, these drugs may be prescribed prophylactically, pre-empting the onset of these ADRs.^81^^,^^82^ Anticholinergic medication targets muscarinic acetylcholine receptors; acetylcholine is important to a range of cognitive functions including memory, attention, and flexibility.^83^^,^^84^ As with clozapine, anticholinergics primarily antagonise the M1 receptor subtype,^85^ potentially disrupting mechanisms key to cognitive processing. The association between anticholinergic prescription and cognition is independent of daily antipsychotic dose. Therefore, this likely reflects a distinct contribution of anticholinergics on cognition, as opposed to capturing our reported antipsychotic dose–cognition association given the higher rates of anticholinergic prescription amongst people on high antipsychotic doses.^86^

We observed several associations between faster genotype-inferred enzyme activity with lower schizophrenia symptom severity. Increased CYP2C19 activity was associated with lower severity of positive symptoms. Associations between CYP2C19 with both schizophrenia severity^14^^,^^87^ and symptom improvement^88^ have been reported although those studies included only people taking clozapine and employed a single, general measure of symptom severity. In the absence of longitudinal symptom scores, we cannot investigate the rather counter-intuitive hypothesis suggested by our data that greater antipsychotic clearance, indexed by CYP2C19 activity, might lead to increased drug effectiveness. CYP2C19 is thought to play a minor part within the clozapine metabolic pathway^89^ but its role in antipsychotic metabolism more widely is poorly documented.^90^ This is an avenue for future studies to follow.

Increased genetically inferred CYP3A5 activity was associated with lower scores on both of the negative symptom dimensions. While associations with the two domains increases confidence in the findings, caution is warranted given that these are moderately correlated (see Fig. 3), and we did not find orthogonal evidence that either of these dimensions were associated with antipsychotic dose. Thus, replication of these associations is required. CYP3A5 is relatively under-examined in antipsychotic research^91^ and its main genotype that leads to slower metabolism (∗1/∗3) is common worldwide but particularly in European populations. Indeed, it has been observed that differences in genotype/phenotype distributions for this enzyme are mainly driven by the inclusion of African ancestries in studies,^92^^,^^93^ as functional CYP3A5 alleles are more common in African countries. Populations from Asian countries also seem to have higher diversity of CYP3A5 alleles than Europe, though research in this continent is still scarce.^94^ Future research aiming to investigate the relevance of CYP3A5 for outcomes in psychiatric populations should therefore aim to include more cohorts from admixed and non-European ancestral makeups.

Finally, we did not observe evidence for an association between CYP1A2 activity score and either the positive or negative schizophrenia symptom dimensions. While a correlation between CYP1A2 activity and symptom severity has been reported,^13^ several differences exist between the methods and sample employed in our own and the previous research. For example, the study by Lesche and colleagues^13^ used a smaller sample, corrected for concomitant medication in the primary analysis, and employed a general measure of phenotype severity (i.e., the total score from the Positive and Negative Syndrome Scale), compared with the dimensional measures used in the present study. Regardless, the observed trends in our data are not consistent with their findings. We also found no association between either the CYP2C9 or CYP2D6 activity scores and any of our phenotype severity dimensions. This is not surprising for CYP2C9, given that it is not widely involved in psychiatric drug clearance. However, CYP2D6 is highly relevant to antipsychotic metabolism, with antipsychotic dose and medication alteration guidelines existing for certain CYP2D6 pharmacogenomic alleles. While an association between CYP2D6 poor metabolism and clinical improvement has been reported,^95^ our own, and previous studies^13^^,^^14^^,^^96^ failed to identify associations between CYP2D6 activity score with symptom severity or other treatment outcomes (i.e., ADRs, medication switching).

This study has several strengths, notably its large sample size in relation to previous research, integration of both medication and pharmacogenomic information, analyses in mixed-medication and clozapine-stratified samples, and finally, adoption of dimensional phenotype severity scores over generalist measures of schizophrenia severity. The final sample size was achieved through incorporating all patients primarily treated with antipsychotic pharmacotherapy, including those with schizophrenia and schizoaffective disorder depressed-type. While diagnostic heterogeneity could bias the results, there is genetic evidence that schizoaffective disorder depressed-type is a sub-type of schizophrenia.^97^ Furthermore, we have statistically controlled for schizophrenia diagnosis and clozapine use, alongside conducting a subgroup analysis of patients prescribed clozapine, widely taken as a proxy measure for a diagnosis of TRS.

The primary limitation of this study is its cross-sectional nature which means we are unable to assess drug treatment response, constraining our ability to make robust inferences about the causal directions for any of the associations. Second, our best fitting CFA model did not include a disorganised dimension, and therefore our study cannot address the possible relationships between this symptom dimension with medication and pharmacogenomic variables. Third, although no individuals were excluded based on ancestry, 95% of the sample reported UK/European as their ethnic background. Cross-cultural differences permeate most aspects of psychiatry, with variation existing in diagnostic criteria, prognosis, therapeutic/prescribing practices, and more.^98^ While ancestrally diverse samples are becoming more common, particularly in larger scale genetics studies, this hasn't always been the case. Therefore, replication of this work across ancestrally diverse samples is required to determine whether these results are not only robust, but generalisable outside of White European backgrounds. Future studies could extend this research by controlling for confounding variables that may also influence symptom severity and cognitive ability in individuals with schizophrenia, such as socio-economic status, comorbid psychiatric disorders, and duration of untreated psychosis. Follow-up confirmatory studies focused on individual genes and/or symptoms would also be desirable to protect against false positive results due to multiple testing, which we controlled via the FDR.

Overall, our findings indicate that poorer cognitive ability in individuals with schizophrenia was associated with the use of clozapine and anticholinergics, alongside high doses of antipsychotic medication. Cognition is an important predictor of schizophrenia functional outcomes.^99^ Therefore, understanding the potentially multifaceted burden of these drugs could help clinicians to minimise the likelihood of cognitive impairment and other poor outcomes during schizophrenia pharmacotherapy. Longitudinal studies, particularly randomised controlled trials, are required to fully understand the role that pharmacotherapy, especially clozapine and anticholinergics, has on cognitive outcomes for those with schizophrenia. However, given the lack of evidence-based guidelines for optimising antipsychotic doses in the maintenance phase of treatment,^100^ addressing potential cognitive impacts might be an actionable target for future interventional research. For example, if information from prescription records (e.g. drug classes and doses) could contribute to identifying patients at high risk of reduced cognition, they could be prioritised for closer monitoring and/or mitigations (e.g., cognitive remediation therapy or avoiding prophylactic anticholinergics).

We also identify associations between the increased activity of certain CYP enzymes and the reduced severity of positive, negative, and cognitive symptoms. Except for CYP1A2 in patients taking clozapine, which we discussed earlier, the mechanisms by which variation within these pharmacogenes could influence schizophrenia severity are unclear. While our results implicate pharmacogenomic variation in antipsychotic pharmacodynamics, an area of psychopharmacology where robust predictors are particularly scarce, further validation of our findings in larger, more diverse samples is required before charting a course from this basic evidence towards improved strategies for patient support and care.

## Contributors

S.E.L. designed the study with input from A.F.P. and M.C.O.D. S.K.L. and D.B.K. processed the CardiffCOGS genomic and phenotypic data. S.K.L. performed statistical and bioinformatics analyses, with input from S.E.L., D.B.K., and A.F.P.; J.T.R.W., M.O. and M.C.O.D. revised the results of all analyses. S.K.L., S.E.L. and A.F.P. draughted the manuscript. All the authors read draughts, contributed to revisions, and approved the final version of the manuscript. A.F.P and S.E.L accessed and verified the underlying data.

## Data sharing statement

Code for reproducing all the main analyses in R is available online at https://locksk.github.io/cogs-symptoms/. Additional scripts for calling pharmacogenomic star alleles and calculating polygenic scores is available online at https://github.com/locksk/symptoms-medication-pgx. To comply with the ethical and regulatory framework of the CardiffCOGS cohort, access to individual-level data requires a collaboration agreement with Cardiff University. Requests to access deidentified datasets, data dictionaries, and other summaries from the CardiffCOGS cohort should be directed to Professor James Walters (waltersjt@cardiff.ac.uk).

## Declaration of interests

MJO, MCOD, and JTRW are supported by a collaborative research grant from Takeda Pharmaceuticals Ltd. for a project unrelated to work presented here. AFP, MJO, MCOD, and JTRW also reported receiving grants from Akrivia Health for a project unrelated to this submission. Takeda and Akrivia Health played no part in the conception, design, implementation, or interpretation of this study.
