# Supplementary material for: Antipsychotic and pharmacogenomic effects on cross-sectional symptom severity and cognitive ability in schizophrenia
Source: eBioMedicine. 2025 May 9;116:105745. doi: 10.1016/j.ebiom.2025.105745 (PMC12139436; doi:10.1016/j.ebiom.2025.105745)
Supplement: Supplementary Material and Tables [file mmc1.docx]

**Supplementary Materials**

**Antipsychotic and pharmacogenomic effects on cross-sectional symptom severity and cognitive ability in schizophrenia**

Siobhan K. Lock^1^ (MSc), Djenifer B. Kappel^1^ (PhD), Michael J. Owen^1^ (PhD), James T.R. Walters^1^ (PhD), Michael C. O'Donovan^1^ (PhD), Antonio F. Pardiñas^1^ (PhD), and Sophie E. Legge^1^ (PhD).

**Affiliations:**

^1^Centre for Neuropsychiatric Genetics and Genomics, Cardiff University, United Kingdom

Hadyn Ellis Building, Maindy Road, Cardiff, United Kingdom, CF24 4HQ

**Corresponding authors:**

Antonio F. Pardiñas: [PardinasA@cardiff.ac.uk](mailto:PardinasA@cardiff.ac.uk) // +44 29206 88407

Hadyn Ellis Building, Maindy Road, Cardiff, United Kingdom, CF24 4HQ

Sophie E. Legge: [LeggeSE8@cardiff.ac.uk](mailto:LeggeSE8@cardiff.ac.uk) // +44 29206 88387

Hadyn Ellis Building, Maindy Road, Cardiff, United Kingdom, CF24 4HQ

**Supplementary Materials: Antipsychotic and pharmacogenomic effects on cross-sectional symptom severity and cognitive ability in schizophrenia.**

[Supplementary Methods 3](#_Toc195688822)

[M1: Imputation of MCCB Test Scores 3](#_Toc195688823)

[M2: Adherence Questionnaire Item 3](#_Toc195688824)

[Supplementary Results 4](#_Toc195688825)

[R1: Associations with the Global Assessment Scale 4](#_Toc195688826)

[R2: Exploring Measures of Premorbid Intelligence 4](#_Toc195688827)

[Comparison of premorbid intelligence measures 4](#_Toc195688828)

[Alternative analyses controlling for premorbid intelligence as estimated by NART 4](#_Toc195688829)

[R3: Sensitivity Analysis - Phenoconversion 5](#_Toc195688830)

[CYP2C19 Phenoconversion 5](#_Toc195688831)

[CYP3A5 Phenoconversion 5](#_Toc195688832)

[CYP1A2 Phenoconversion 5](#_Toc195688833)

[Supplementary Tables 6](#_Toc195688834)

[Supplementary Table 1. CYP Pharmacogenomic star alleles and their respective activity scores. 6](#_Toc195688835)

[Supplementary Table 2. Antipsychotic use in CardiffCOGS 7](#_Toc195688836)

[Supplementary Table 3. CYP Pharmacogenomic star allele frequencies in CardiffCOGS 8](#_Toc195688837)

[Supplementary Table 4. Summary of CFA results 9](#_Toc195688838)

[Supplementary Table 5. Medication model controlling for diagnosis 10](#_Toc195688839)

[Supplementary Table 6. Unadjusted estimates for pharmacogenomic variables in total sample. 11](#_Toc195688840)

[Supplementary Table 7. Unadjusted estimates for pharmacogenomic variables in the subgroup of participants prescribed clozapine. 12](#_Toc195688841)

[Supplementary Table 8. Sensitivity analyses accounting for potential fluoxetine-induced CYP2C19 phenoconversion. 13](#_Toc195688842)

[Supplementary Table 9. Sensitivity analyses accounting for potential carbamazepine-induced CYP3A5 phenoconversion. 14](#_Toc195688843)

[Supplementary Table 10. Sensitivity analyses accounting for potential smoking-induced CYP1A2 phenoconversion. 15](#_Toc195688844)

[Supplementary Table 11. Alternative Analyses using NART IQ as a proxy of premorbid intelligence 16](#_Toc195688845)

[STrengthening the REporting of Genetic Association studies (STREGA) reporting recommendations, extended from STROBE Statement 17](#_Toc195688846)

[Supplementary References 22](#_Toc195688847)

## Supplementary Methods

### M1: Imputation of MCCB Test Scores

Scores on the MATRICS Consensus Cognitive Battery (MCCB) were first standardized against unaffected controls to create z-scores, and then imputed in line with the recommended procedures from the MCCB handbook^1^, equation supplied below:

$$\hat{Y}_{di}= \bar{Y}_{d+}+ \bar{Y}_{+i}- \bar{Y}_{++}$$

$Y_{di}$ refers to the score for person *i* on test *d*, and the notation + refers to the average of the index *i* or *d*. Therefore, $\bar{Y}_{d+}$ refers to the mean score for test *d* across all cases, $\bar{Y}_{+i}$ refers to the mean score of all tests for individual *i*, and $\bar{Y}_{++}$ is the mean score for all tests across all individuals. This was modified to make use of existing, test-relevant information, where possible. When test scores utilised information from across multiple trials (e.g., CPT, HVLT, BVMT), scores from completed trials (e.g., when participants had completed one or two trials out of 3) were used to impute the missing score.

For HVLT or BVMT, test scores are total scores from across the three trials. When no scores are available, the total score is imputed based on the original procedures. When participants completed one or two trials, the formula was modified so that only participants with matched scores on the non-missing trial were used to calculate the average test score ($\bar{Y}_{d+}$). For example, participants scoring 3, 5, NA, were matched with complete cases who scored 8 (i.e., 3 + 5) across the first two trials; $\bar{Y}_{d+}$will then be calculated based on these participants.

For CPT, the test score is the average from across the three trials. When no scores are available, the mean score is imputed based on the original procedures. If one or two scores are available, imputation follows the altered procedures but including only CPT scores. Therefore, $\bar{Y}_{d+}$ becomes the mean score of that CPT trial for all cases, $\bar{Y}_{+i}$ becomes the mean score of all completed CPT trials for individual *i*, and $\bar{Y}_{++}$ becomes the mean score of all CPT trials for all individuals. The mean score is then calculated for individuals with missing trials using both completed and imputed trial scores.

When domain scores are calculated based on scores from multiple tasks (e.g., WM, SoP) the procedures are adapted as follows:

For Working Memory (WM), domain scores are calculated based on scores from two tasks (WMS-III Spatial Span and the Letter-Number Span). If scores from both tasks are missing, then impute the domain score itself based on the original procedure. If only one task is missing, impute the missing test score and calculate the domain score based on the completed and imputed test scores.

For Speed of Processing (SoP), domain scores are calculated based on scores from three tasks (BACS: Symbol Coding, Category Fluency: Animal Naming, Trail Making Test: Part A). If scores from two or three tasks are missing, then impute the domain score itself based on the original procedure. If only one task is missing, impute the missing test score and calculate the domain score based on the completed and imputed test scores.

### M2: Adherence Questionnaire Item

Adherence to current medication was measured on an ordinal scale as described below. Participants were rated according to the most appropriate statement:

1. Doesn't take medication.
2. Takes medication only rarely (1-2/week).
3. Misses over 50% doses.
4. Misses doses every week.
5. Rarely misses doses.
6. Medication supervised and takes as prescribed.

Responses were dichotomised due to low numbers of ratings in certain categories. Participants who missed doses every week, or more frequently (0-3) were assigned as non-adherent. Those who reported rarely missed doses, or were medication supervised (4-5) were assigned as adherent.

## Supplementary Results

### R1: Associations with the Global Assessment Scale

Four factor structures were fit to determine which best represented the data as described in Supplementary Table 4. Scores for all latent variables were significantly associated with current Global Assessment Scale ratings in models that controlled for age and sex. In all instances, increased symptom severity was associated with reduced scores on the Global Assessment Scale. This manifested as inverse associations between the positive (β = -0.54; 95%CI, -0.62 to -0.46; *p* = 6x10⁻³⁷), diminished expressivity (β = -0.416; 95%CI, -0.5 to -0.33; *p* = 2x10⁻²¹), reduced motivation and pleasure (β = -0.544; 95%CI, -0.62 to -0.47; *p* = 4x10⁻³⁹), and depression/suicide dimensions (β = -0.543; 95%CI, -0.62 to -0.47; *p* = 3x10⁻³⁷) with Global Assessment Scale scores. The cognition dimension was positively associated with GAS (β = 0.174; 95%CI, 0.09 to 0.26; *p* = 3x10⁻⁵), with higher ratings across the Global Assessment Scale and the cognition dimension both reflecting improved functioning.

### R2: Exploring Measures of Premorbid Intelligence

#### Comparison of premorbid intelligence measures

Premorbid intelligence as estimated by the NART had a stronger association with the cognitive ability dimension (β = 0.517; 95%CI, 0.45 to 0.58; p = 5x10⁻⁴⁷), compared to the intelligence PGS (β = 0.097; 95%CI, 0.01 to 0.19; p = 0.034) and educational attainment PGS (β = 0.115; 95%CI, 0.03 to 0.2; p = 0.012).

However, we also found that premorbid intelligence, as estimated by the NART, was inversely associated with chlorpromazine-equivalent antipsychotic dose (β = -0.131; 95%CI, -0.21 to -0.05; p = 0.002) whereas there was no such association with dose for either intelligence PGS (β = -0.034; 95%CI, -0.13 to 0.06; p = 0.495) or educational attainment PGS (β = -0.016; 95%CI, -0.11 to 0.08; p = 0.75). The above results controlled for age and sex; for models including genetic predictors (i.e., intelligence and education PGS) we also covaried for genetic principal components one to five.

The results could indicate that participants with lower premorbid intelligence scores from the NART were prescribed higher antipsychotic doses. An alternative interpretation is that performance on the NART was affected by the participant’s antipsychotic dose (i.e. sedation effects) and therefore, might not fully reflect pre-morbid intelligence in this sample^2^. As we were not able to distinguish between these potential confounding effects, we selected to use both intelligence and educational PGS as the estimates of premorbid intelligence in our primary analyses. However, we have conducted secondary analyses controlling for the NART, shown below.

#### Alternative analyses controlling for premorbid intelligence as estimated by NART

This secondary analysis controls for premorbid intelligence as estimated by the NART, instead of PGS for intelligence and educational attainment, in analyses where the cognitive ability dimension is the outcome. Results are presented in Supplementary Table 11. NART data was missing for some participants in the full sample (N = 27) and those prescribed clozapine (N = 13); hence, these analyses have a lower sample size than those reported in the main text.

The associations between antipsychotic dose, clozapine use, and anticholinergic use with cognitive ability remained significant when including NART in the models, despite an attenuation in the strength of effect sizes and *p*-values. However, in the pharmacogenomic model including only patients prescribed clozapine, covarying for NART reduced the effect size for the associations of cognitive ability with anticholinergic use and CYP1A2 activity score, rendering their *p*-values non-significant.

### R3: Sensitivity Analysis – Phenoconversion

We observed associations between genotype-inferred activity of three enzymes with schizophrenia phenotype dimensions. Based on the Food and Drug Administration’s (FDA) table of CYP Enzyme- and Transporter System-Based Clinical Substrates, Inhibitors, or Inducers^3^, we identified three drugs within the CardiffCOGS sample that could influence the activity of these enzymes. These drugs include the CYP2C19 strong inhibitor fluoxetine (N = 34), the CYP3A5 strong inducer carbamazepine (N = 5), and the CYP1A2 moderate inducer cigarette smoking (N = 305). We are however unable to account for other drugs and factors not reported in our study (e.g., oral contraceptives, dietary factors, disease, and inflammation) that may further influence enzyme activity^4,5^.

#### CYP2C19 Phenoconversion

There was no evidence of a significant interaction between the CYP2C19 activity score and fluoxetine use (β = -0.123; 95%CI, -0.41 to 0.17; *p* = 0.41). However, CYP2C19 activity remained significantly associated with the positive symptom dimension (β = -0.09; 95%CI, -0.17 to -0.01; *p* = 0.037). Fluoxetine use was also inversely associated with positive dimension scores (β = -0.363; 95%CI, -0.71 to -0.02; *p* = 0.04). A phenoconversion-corrected CYP2C19 activity score was not associated with the positive symptom dimension (β = -0.022; 95%CI, -0.1 to 0.06; *p* = 0.6).

#### CYP3A5 Phenoconversion

The number of individuals taking carbamazepine was very low (N = 5); therefore, we were unable to model an interaction between CYP3A5 with carbamazepine use. Equally, we did not calculate phenoconversion-corrected activity scores as all carbamazepine users had null function alleles – thus, their activity scores would have been unchanged (i.e., 0 x 1.5 = 0). The associations between CYP3A5 activity remained with both the diminished expressivity (β = -0.106; 95%CI, -0.19 to -0.02; *p* = 0.01) and reduced motivation and pleasure dimensions (β = -0.1; 95%CI, -0.18 to -0.02; *p* = 0.018) after accounting for carbamazepine use through a covariate, which was itself not associated with either symptom dimension.

#### CYP1A2 Phenoconversion

We saw no significant interaction between the CYP1A2 activity score and cigarette use (β = -0.066; 95%CI, -0.33 to 0.19; *p* = 0.62). Cigarette smoking was inversely associated with cognition (β = -0.29; 95%CI, -0.54 to -0.04; *p* = 0.026); however, the association between CYP1A2 activity and cognition weakened (β = 0.19; 95%CI, -0.01 to 0.39; *p* = 0.06). Finally, we observed no significant association between the phenoconversion-corrected CYP1A2 activity scores with cognition (β = -0.022; 95%CI, -0.15 to 0.11; *p* = 0.74).

The *p* value for the association between CYP1A2 activity score and cognition became larger after controlling for smoking status, suggesting that part of the variance in cognition explained by CYP1A2 is due to the inductive effect of cigarette smoking. However, information regarding patient smoking status was absent for just under 10% of the original subgroup (N = 18). The attenuation of the CYP1A2-cognition association could be explained in part by this loss of statistical power. We note that the negative association between cigarette smoking, and cognition is somewhat contrary to expectations. Cigarette smoke is a known CYP1A2 inducer, binding with aryl hydrocarbon receptors and resulting in increased enzyme activity^6^. However, chronic smoking is also robustly associated with cognitive impairment^7^. Therefore, smoking may be exerting dual, contradictory effects on cognition in our sample; a larger, inverse direct effect, and a more modest indirect, positive effect through induction of CYP1A2 activity.

## Supplementary Tables

### Supplementary Table 1. CYP Pharmacogenomic star alleles and their respective activity scores.

| **Enzyme** | **Allele** | **Function** | **Activity Score** | **Source** |
| --- | --- | --- | --- | --- |
| CYP1A2 | 1A | Normal | 1 | Lesche et al., 2020^8^ |
| CYP1A2 | 1C | Decreased | 0.5 | Saiz-Rodriguez et al., 2019^9^ |
| CYP1A2 | 1F | Increased | 1.5 | Lesche et al., 2020^8^ |
| CYP1A2 | 1K | Decreased | 0.5 | N/A |
| CYP2D6 | 1 | Normal | 1 | PyPGx^10^ |
| CYP2D6 | 2 | Normal | 1 | PyPGx^10^ |
| CYP2D6 | 4 | Null | 0 | PyPGx^10^ |
| CYP2D6 | 10 | Decreased | 0.25 | PyPGx^10^ |
| CYP2D6 | 17 | Decreased | 0.5 | PyPGx^10^ |
| CYP2D6 | 28 | Uncertain | N/A | PyPGx^10^ |
| CYP2D6 | 35 | Normal | 1 | PyPGx^10^ |
| CYP2D6 | 41 | Decreased | 0.5 | PyPGx^10^ |
| CYP2D6 | 59 | Decreased | 0.5 | PyPGx^10^ |
| CYP2D6 | 117 | Uncertain | N/A | PyPGx^10^ |
| CYP3A5 | 1 | Normal | 1 | PharmGKB Gene-Specific Information Tables^11^ |
| CYP3A5 | 3 | Null | 0 | PharmGKB Gene-Specific Information Tables^11^ |
| CYP2C19 | 1 | Normal | 1 | Lesche et al., 2020^8^ |
| CYP2C19 | 2 | Null | 0 | Lesche et al., 2020^8^ |
| CYP2C19 | 8 | Null | 0 | PharmGKB Gene-Specific Information Tables^11^ |
| CYP2C19 | 15 | Normal | 1 | PharmGKB Gene-Specific Information Tables^11^ |
| CYP2C19 | 17 | Increased | 1.5 | Lesche et al., 2020^8^ |
| CYP2C9 | 1 | Normal | 1 | PyPGx^10^ |
| CYP2C9 | 2 | Decreased | 0.5 | PyPGx^10^ |
| CYP2C9 | 3 | Null | 0 | PyPGx^10^ |
| CYP2C9 | 9 | Normal | 1 | PyPGx^10^ |
| CYP2C9 | 11 | Decreased | 0.5 | PyPGx^10^ |
| CYP2C9 | 12 | Decreased | 0.5 | PyPGx^10^ |

Supplementary Table 1. CYP pharmacogenomic star alleles called in the CardiffCOGS sample. Star allele function, and corresponding activity scores are listed, alongside sources for mapping of star alleles to activity scores. A complete list of variants that qualify as star alleles in PyPGx^10^ is available at <https://github.com/sbslee/pypgx/tree/master/pypgx/api/data>

### Supplementary Table 2. Antipsychotic use in CardiffCOGS

| **Antipsychotic** | **N** |
| --- | --- |
| Amisulpride | 50 |
| Aripiprazole | 74 |
| Chlorpromazine | 8 |
| Clozapine | 215 |
| Flupenthixol | 60 |
| Fluphenazine | 6 |
| Haloperidol | 15 |
| Olanzapine | 103 |
| Paliperidone | < 5 |
| Pimozide | < 5 |
| Pipotiazine | < 5 |
| Quetiapine | 52 |
| Risperidone | 81 |
| Sulpiride | 10 |
| Trifluoperazine | < 5 |
| Zuclopenthixol | 13 |

Supplementary Table 2. Frequency of currently used antipsychotics in the CardiffCOGS sample.

### Supplementary Table 3. CYP Pharmacogenomic star allele frequencies in CardiffCOGS

| **Gene** | **Star Allele** | **Total** | **Frequency** |
| --- | --- | --- | --- |
| *CYP1A2* | *1A | 403 | 0.344 |
|  | *1C*1F | 16 | 0.014 |
|  | *1F | 748 | 0.639 |
|  | *1K | 3 | 0.003 |
| *CYP2D6* | *1 | 556 | 0.475 |
|  | *10 | 10 | 0.009 |
|  | *2 | 181 | 0.155 |
|  | *35 | 50 | 0.043 |
|  | *4 | 246 | 0.210 |
|  | *41 | 110 | 0.094 |
|  | *117 | 2 | 0.002 |
|  | *17 | 5 | 0.004 |
|  | *28 | 5 | 0.004 |
|  | *59 | 5 | 0.004 |
| *CYP3A5* | *1 | 67 | 0.057 |
|  | *3 | 1103 | 0.943 |
| *CYP2C19* | *1 | 740 | 0.632 |
|  | *15 | 1 | 0.001 |
|  | *17 | 255 | 0.218 |
|  | *2 | 172 | 0.147 |
|  | *8 | 2 | 0.002 |
| *CYP2C9* | *1 | 946 | 0.809 |
|  | *2 | 135 | 0.115 |
|  | *3 | 78 | 0.067 |
|  | *11 | 8 | 0.007 |
|  | *12 | 2 | 0.002 |

Supplementary Table 3. Total allele count, and allele frequency for CYP pharmacogenomic star alleles found in the CardiffCOGS sample.

### Supplementary Table 4. Summary of CFA results

| Latent Factor | Variable | Mean | SD | Min | Max | M1 | M2 | M3 | M4 |
| --- | --- | --- | --- | --- | --- | --- | --- | --- | --- |
| Positive | SAPS Global Hallucinations | 1.338 | 1.852 | 0.00 | 5.00 | 0.769 | 0.874 | 0.681 | 0.858 |
| Positive | SAPS Global Delusions | 1.255 | 1.595 | 0.00 | 5.00 | 0.750 | 0.659 | 0.847 | 0.672 |
| Diminished Expressivity | SANS Global Affective Flattening | 1.383 | 1.435 | 0.00 | 5.00 | 0.872 | 0.880 | 0.859 | 0.867 |
| Diminished Expressivity | SANS Global Alogia | 1.125 | 1.475 | 0.00 | 5.00 | 0.771 | 0.764 | 0.782 | 0.775 |
| Reduced Motivation and Pleasure | SANS Global Avolition/Apathy | 1.781 | 1.327 | 0.00 | 5.00 | 0.840 | 0.839 | 0.847 | 0.846 |
| Reduced Motivation and Pleasure | SANS Global Anhedonia/Asociality | 1.853 | 1.516 | 0.00 | 5.00 | 0.760 | 0.762 | 0.754 | 0.756 |
| Disorganised | SAPS Global Positive Thought Disorder | 0.583 | 1.106 | 0.00 | 5.00 | 0.492 | *EXCL* | 0.493 | *EXCL* |
| Disorganised | SANS Inappropriate Affect | 0.080 | 0.448 | 0.00 | 4.00 | 0.526 | *EXCL* | 0.525 | *EXCL* |
| Suicide and Depression | CDSS Suicide item | 0.157 | 0.417 | 0.00 | 3.00 | 0.913 | 0.904 | *EXCL* | *EXCL* |
| Suicide and Depression | CDSS Depression item | 0.533 | 0.747 | 0.00 | 3.00 | 0.808 | 0.817 | *EXCL* | *EXCL* |
| Cognitive ability | MATRICS Problem Solving task (mazes)* | -1.513 | 1.294 | -3.99 | 1.17 | 0.653 | 0.654 | 0.652 | 0.653 |
| Cognitive ability | MATRICS Verbal Learning task (HVLT)* | -2.447 | 1.521 | -6.38 | 1.59 | 0.743 | 0.742 | 0.744 | 0.742 |
| Cognitive ability | MATRICS Visual Learning task (BVMT)* | -1.646 | 1.222 | -3.92 | 1.52 | 0.707 | 0.708 | 0.708 | 0.709 |
| Cognitive ability | MATRICS Attention (CPT)* | -1.438 | 1.228 | -5.52 | 2.25 | 0.733 | 0.732 | 0.732 | 0.730 |
| Cognitive ability | MATRICS Processing Speed* | -1.951 | 1.104 | -5.45 | 1.32 | 0.834 | 0.835 | 0.835 | 0.836 |
| Cognitive ability | MATRICS Working Memory* | -1.641 | 1.193 | -4.59 | 1.56 | 0.831 | 0.831 | 0.832 | 0.832 |
| Goodness of Fit Measures | |  |  |  |  |  |  |  |  |
| CFI | |  |  |  |  | 0.996 | 0.997 | 0.996 | 0.995 |
| RMSEA | |  |  |  |  | 0.020 | 0.019 | 0.024 | 0.028 |
| SRMR | |  |  |  |  | 0.053 | 0.043 | 0.050 | 0.044 |

Supplementary Table 4. Summary of full CFA results. Descriptive statistics are listed alongside factor loadings for each variable onto its latent factors across the four considered models (M1 – M4). Key fit statistics are listed below, with the best fit highlighted in red. M1 represents the maximal model with all variables included. M2 represents the maximal model without the disorganised variables. M3 represents the maximal model without the suicide/depression variables (i.e., the 5-factor model as based on Legge et al., 2021). M4 represents the maximal model with neither the suicide/depression nor disorganised factors. EXCL = excluded; CFI = Comparative Fit Index; RMSEA = Root Mean Square Error of Approximation; SRMR = Standardised Root Mean Square Residual. *MATRICS variables were imputed and standardised against unaffected controls.

### Supplementary Table 5. Medication model controlling for diagnosis

|  | Positive | | Diminished Expressivity | | Reduced Motivation & Pleasure | | Depression & Suicide | | Cognition | |
| --- | --- | --- | --- | --- | --- | --- | --- | --- | --- | --- |
| **Predictors** | **β (SE)** | ***p*(FDR)** | **β (SE)** | ***p*(FDR)** | **β (SE)** | ***p*(FDR)** | **β (SE)** | ***p*(FDR)** | **β (SE)** | ***p*(FDR)** |
| CPZ-eq Antipsychotic Dose (mg/day) | 0.149  (0.04) | 6x10⁻⁴  (0.003) | 0.046  (0.04) | 0.28  (0.28) | 0.063  (0.04) | 0.146  (0.182) | 0.116  (0.04) | 0.006  (0.011) | -0.122  (0.04) | 0.001  (0.003) |
| Clozapine (Yes) | -0.051  (0.09) | 0.565  (0.692) | 0.424  (0.09) | 2x10⁻⁶  (5x10⁻⁶) | 0.25  (0.09) | 0.005  (0.009) | 0.035  (0.09) | 0.692  (0.692) | -0.504  (0.08) | 2x10⁻¹⁰  (1x10⁻⁹) |
| Anticholinergic (Yes) | 0.078  (0.12) | 0.506  (0.506) | 0.221  (0.12) | 0.057  (0.087) | 0.267  (0.12) | 0.023  (0.057) | 0.211  (0.12) | 0.069  (0.087) | -0.343  (0.1) | 9x10⁻⁴  (0.004) |
| Adherent (Yes) | -0.5  (0.19) | 0.007  (0.037) | -0.236  (0.18) | 0.198  (0.198) | -0.368  (0.19) | 0.048  (0.088) | -0.355  (0.18) | 0.053  (0.088) | 0.261  (0.16) | 0.109  (0.136) |
| Schizophrenia Diagnosis | -0.114  (0.11) | 0.314  (0.487) | -0.081  (0.11) | 0.468  (0.487) | -0.204  (0.11) | 0.073  (0.182) | -0.489  (0.11) | 1x10⁻⁵ (7x10⁻⁵) | 0.069  (0.1) | 0.487  (0.487) |
| Schizophrenia PGS | -0.042  (0.04) | 0.329  (0.549) | 0.028  (0.04) | 0.5  (0.625) | -0.007  (0.04) | 0.864  (0.864) | -0.065  (0.04) | 0.12  (0.3) | -0.099  (0.04) | 0.009  (0.045) |
| Intelligence PGS |  |  |  |  |  |  |  |  | 0.076  (0.04) | 0.085  (0.085) |
| Educational Attainment PGS |  |  |  |  |  |  |  |  | 0.129  (0.04) | 0.003  (0.003) |
| Age | -0.049  (0.04) | 0.251  (0.418) | 0.06  (0.04) | 0.152  (0.38) | 0.034  (0.04) | 0.419  (0.524) | -0.004  (0.04) | 0.929  (0.929) | -0.376  (0.04) | 3x10⁻²²  (2x10⁻²¹) |
| Sex (Female) | 0.033  (0.09) | 0.71  (0.71) | -0.19  (0.09) | 0.031  (0.154) | -0.138  (0.09) | 0.12  (0.3) | 0.083  (0.09) | 0.347  (0.578) | 0.033  (0.08) | 0.675  (0.71) |
| PC1 | -0.022  (0.04) | 0.601  (0.769) | 0.012  (0.04) | 0.783  (0.783) | -0.021  (0.04) | 0.615  (0.769) | -0.047  (0.04) | 0.256  (0.769) | 0.027  (0.04) | 0.471  (0.769) |
| PC2 | -0.033  (0.04) | 0.42  (0.573) | 0.027  (0.04) | 0.513  (0.573) | 0.023  (0.04) | 0.573  (0.573) | -0.051  (0.04) | 0.21  (0.525) | -0.048  (0.04) | 0.187  (0.525) |
| PC3 | -0.061  (0.04) | 0.136  (0.679) | 0.009  (0.04) | 0.826  (0.914) | 0.009  (0.04) | 0.828  (0.914) | 0.004  (0.04) | 0.914  (0.914) | -0.018  (0.04) | 0.608  (0.914) |
| PC4 | 0.003  (0.04) | 0.948  (0.948) | -0.031  (0.04) | 0.452  (0.878) | -0.016  (0.04) | 0.703  (0.878) | -0.022  (0.04) | 0.587  (0.878) | 0.041  (0.04) | 0.26  (0.878) |
| PC5 | -0.034  (0.04) | 0.404  (0.574) | 0.062  (0.04) | 0.127  (0.394) | 0.058  (0.04) | 0.158  (0.394) | 0.023  (0.04) | 0.574  (0.574) | 0.023  (0.04) | 0.53  (0.574) |
|  |  |  |  |  |  |  |  |  |  |  |
| R2 | 0.047 | | 0.069 | | 0.047 | | 0.072 | | 0.275 | |
| Adjusted R2 | 0.025 | | 0.048 | | 0.025 | | 0.051 | | 0.256 | |
| N | 585 | | 585 | | 585 | | 585 | | 585 | |

Supplementary Table 5. Associations between medication variables with schizophrenia symptom severity and cognitive ability dimensions in CardiffCOGS, controlling for diagnosis of schizophrenia or schizoaffective disorder depressed-type. Standardised regression estimates are reported. CPZ-eq = chlorpromazine-equivalent; SE = Standard Error; PGS = Polygenic Score; PC = Genetic Principal Component.

### Supplementary Table 6. Unadjusted estimates for pharmacogenomic variables in total sample.

|  | Positive | | Diminished Expressivity | | Reduced Motivation & Pleasure | | Depression & Suicide | | Cognition | |
| --- | --- | --- | --- | --- | --- | --- | --- | --- | --- | --- |
| **Enzyme** | **r** | ***p*** | **r** | ***p*** | **r** | ***p*** | **r** | ***p*** | **r** | ***P*** |
| CYP1A2 Activity Score | -0.052 | 0.209 | -0.050 | 0.231 | -0.026 | 0.525 | -0.044 | 0.290 | 0.092 | 0.026 |
| CYP2D6 Activity Score | 0.004 | 0.919 | 0.048 | 0.245 | 0.048 | 0.248 | 0.014 | 0.744 | -0.063 | 0.128 |
| CYP3A5 Activity Score | -0.046 | 0.265 | -0.098 | 0.018 | -0.095 | 0.022 | -0.040 | 0.333 | 0.025 | 0.548 |
| CYP2C19 Activity Score | -0.114 | 0.006 | -0.003 | 0.937 | -0.045 | 0.281 | -0.085 | 0.041 | 0.017 | 0.687 |
| CYP2C9 Activity Score | -0.050 | 0.233 | -0.029 | 0.486 | -0.009 | 0.821 | -0.046 | 0.268 | -0.026 | 0.533 |

Supplementary Table 6. Unadjusted estimates for pharmacogenomic variables against schizophrenia phenotype dimensions. Table shows Pearson’s correlation coefficient and *p* value for each dimension-enzyme pairing.

### Supplementary Table 7. Unadjusted estimates for pharmacogenomic variables in the subgroup of participants prescribed clozapine.

|  | Positive | | Diminished Expressivity | | Reduced Motivation & Pleasure | | Depression & Suicide | | Cognition | |
| --- | --- | --- | --- | --- | --- | --- | --- | --- | --- | --- |
| **Enzyme** | **r** | ***p*** | **r** | ***p*** | **r** | ***p*** | **r** | ***p*** | **r** | ***P*** |
| CYP1A2 Activity Score | -0.055 | 0.422 | -0.102 | 0.137 | -0.035 | 0.613 | -0.040 | 0.561 | 0.205 | 0.002 |
| CYP2D6 Activity Score | -0.014 | 0.844 | 0.114 | 0.094 | 0.102 | 0.135 | 0.025 | 0.711 | -0.110 | 0.107 |
| CYP3A5 Activity Score | -0.084 | 0.221 | -0.113 | 0.100 | -0.105 | 0.125 | -0.123 | 0.072 | 0.068 | 0.319 |
| CYP2C19 Activity Score | -0.066 | 0.337 | 0.017 | 0.806 | 0.009 | 0.890 | 0.038 | 0.575 | -0.026 | 0.705 |
| CYP2C9 Activity Score | -0.044 | 0.517 | -0.186 | 0.006 | -0.142 | 0.037 | -0.138 | 0.043 | 0.089 | 0.195 |

Supplementary Table 7. Unadjusted estimates for pharmacogenomic variables against Schizophrenia Phenotype Dimensions. Table shows Pearson’s correlation coefficient and *p* value for each dimension-enzyme pairing dimensions in CardiffCOGS participants currently prescribed clozapine.

### Supplementary Table 8. Sensitivity analyses accounting for potential fluoxetine-induced CYP2C19 phenoconversion.

|  | Positive  CYP2C19 – Fluoxetine Interaction | | Positive  CYP2C19 Phenoconversion-Corrected Activity Scores | |
| --- | --- | --- | --- | --- |
| **Predictors** | **β (SE)** | ***p*** | **β (SE)** | ***p*** |
| CPZ-eq Antipsychotic Dose (mg/day) | 0.135 (0.04) | 0.002 | 0.145 (0.04) | 7x10⁻⁴ |
| Clozapine (Yes) | -0.056 (0.09) | 0.522 | -0.058 (0.09) | 0.515 |
| Anticholinergic (Yes) | 0.081 (0.12) | 0.487 | 0.077 (0.12) | 0.513 |
| CYP2C19 Activity Score | -0.09 (0.04) | 0.037 |  |  |
| Fluoxetine (Yes) | -0.363 (0.18) | 0.04 |  |  |
| CYP2C19-Fluoxetine interaction | -0.123 (0.15) | 0.408 |  |  |
| Phenoconversion-Corrected CYP2C19 Activity Score |  |  | -0.022 (0.04) | 0.6 |
| Adherent (Yes) | -0.517 (0.19) | 0.005 | -0.491 (0.19) | 0.009 |
| Schizophrenia PGS | -0.045 (0.04) | 0.289 | -0.042 (0.04) | 0.321 |
| Age | -0.056 (0.04) | 0.186 | -0.048 (0.04) | 0.262 |
| Sex (Female) | 0.07 (0.09) | 0.419 | 0.05 (0.09) | 0.566 |
| PC1 | -0.027 (0.04) | 0.525 | -0.025 (0.04) | 0.549 |
| PC2 | -0.019 (0.04) | 0.643 | -0.036 (0.04) | 0.392 |
| PC3 | -0.053 (0.04) | 0.196 | -0.061 (0.04) | 0.139 |
| PC4 | 0.003 (0.04) | 0.95 | 0.003 (0.04) | 0.949 |
| PC5 | -0.034 (0.04) | 0.401 | -0.033 (0.04) | 0.428 |
|  |  |  |  |  |
| R2 | 0.062 | | 0.045 | |
| Adjusted R2 | 0.037 | | 0.024 | |
| N | 585 | | 585 | |

Supplementary Table 8. Sensitivity analyses to account for the potential of fluoxetine to inhibit CYP2C19 activity. This is accounted for through a control (fluoxetine yes/no) covariate, or by inclusion of a phenoconversion activity score (activity score * 0, if participant reports fluoxetine). Sensitivity analyses were performed only in the positive dimension where an association with CYP2C19 was previously observed.

### Supplementary Table 9. Sensitivity analyses accounting for potential carbamazepine-induced CYP3A5 phenoconversion.

|  | Diminished Expressivity  CYP3A5 – Carbamazepine Covariate | | Reduced Motivation & Pleasure  CYP3A5 – Carbamazepine Interaction | |
| --- | --- | --- | --- | --- |
| **Predictors** | **β (SE)** | ***p*** | **β (SE)** | ***p*** |
| CPZ-eq Antipsychotic Dose (mg/day) | 0.043 (0.04) | 0.303 | 0.056 (0.04) | 0.192 |
| Clozapine (Yes) | 0.428 (0.09) | 1x10⁻⁶ | 0.242 (0.09) | 0.006 |
| Anticholinergic (Yes) | 0.236 (0.12) | 0.041 | 0.284 (0.12) | 0.015 |
| CYP3A5 Activity Score | -0.106 (0.04) | 0.01 | -0.1 (0.04) | 0.018 |
| Carbamazepine (Yes) | 0.644 (0.44) | 0.142 | 0.597 (0.44) | 0.18 |
| Adherent (Yes) | -0.229 (0.18) | 0.21 | -0.359 (0.19) | 0.053 |
| Schizophrenia PGS | 0.029 (0.04) | 0.492 | -0.006 (0.04) | 0.88 |
| Age | 0.065 (0.04) | 0.12 | 0.04 (0.04) | 0.345 |
| Sex (Female) | -0.181 (0.09) | 0.034 | -0.109 (0.09) | 0.21 |
| PC1 | 0.024 (0.04) | 0.573 | -0.015 (0.04) | 0.732 |
| PC2 | 0.02 (0.04) | 0.631 | 0.016 (0.04) | 0.703 |
| PC3 | 0.02 (0.04) | 0.629 | 0.018 (0.04) | 0.659 |
| PC4 | -0.018 (0.04) | 0.663 | -0.003 (0.04) | 0.937 |
| PC5 | 0.064 (0.04) | 0.112 | 0.063 (0.04) | 0.124 |
|  |  |  |  |  |
| R2 | 0.083 | | 0.054 | |
| Adjusted R2 | 0.060 | | 0.031 | |
| N | 585 | | 585 | |

Supplementary Table 9. Sensitivity analysis to account for the potential of carbamazepine to induce CYP3A5 activity. This is accounted for through a control (carbamazepine yes/no) covariate for both symptom dimensions (i.e., diminished expressivity, reduced motivation and pleasure) where an association with CYP3A5 activity score was previously observed.

### Supplementary Table 10. Sensitivity analyses accounting for potential smoking-induced CYP1A2 phenoconversion.

|  | Cognitive (Clozapine Subgroup)  CYP1A2 – Cigarette Interaction | | Cognitive (Clozapine Subgroup)  CYP1A2 Phenoconversion-Corrected Activity Scores | |
| --- | --- | --- | --- | --- |
| **Predictors** | **β (SE)** | ***p*** | **β (SE)** | ***p*** |
| CPZ-eq Antipsychotic Dose (mg/day) | -0.133 (0.06) | 0.038 | -0.147 (0.06) | 0.024 |
| Anticholinergic (Yes) | -0.352 (0.17) | 0.043 | -0.375 (0.18) | 0.034 |
| CYP1A2 Activity Score | 0.19 (0.1) | 0.06 |  |  |
| Cigarettes (Yes) | -0.29 (0.13) | 0.026 |  |  |
| CYP1A2-Smoking interaction | -0.066 (0.13) | 0.618 |  |  |
| Phenoconversion-Corrected CYP1A2 Activity Score |  |  | -0.022 (0.06) | 0.739 |
| Adherent (Yes) | 0.206 (0.44) | 0.644 | 0.207 (0.45) | 0.648 |
| Schizophrenia PGS | -0.092 (0.07) | 0.165 | -0.102 (0.07) | 0.133 |
| Intelligence PGS | 0.147 (0.08) | 0.078 | 0.14 (0.08) | 0.094 |
| Educational Attainment PGS | 0.032 (0.08) | 0.704 | 0.074 (0.08) | 0.367 |
| Age | -0.401 (0.06) | 8x10⁻¹⁰ | -0.405 (0.06) | 9x10⁻¹⁰ |
| Sex (Female) | 0.029 (0.13) | 0.823 | 0.066 (0.13) | 0.617 |
| PC1 | 0.094 (0.06) | 0.143 | 0.09 (0.06) | 0.165 |
| PC2 | 0.138 (0.13) | 0.293 | 0.178 (0.13) | 0.18 |
| PC3 | -0.429 (0.25) | 0.091 | -0.488 (0.26) | 0.058 |
| PC4 | 0.403 (0.25) | 0.102 | 0.482 (0.25) | 0.054 |
| PC5 | -0.018 (0.09) | 0.848 | 0.008 (0.09) | 0.931 |
|  |  |  |  |  |
| R2 | 0.339 | | 0.301 | |
| Adjusted R2 | 0.284 | | 0.251 | |
| N | 197 | | 197 | |

Supplementary Table 10. Sensitivity analysis to account for the potential of smoking to influence CYP1A2 activity. This is accounted for through a control (smoking yes/no) covariate, or by inclusion of a phenoconversion activity score (activity score * 1.5 if participant is a smoker). Sensitivity analyses were performed only in the cognitive dimension where an association with CYP1A2 activity score was previously observed.

### Supplementary Table 11. Alternative Analyses using NART IQ as a proxy of premorbid intelligence

|  | Medication Model | | Medication & Pharmacogenomic Model | | Medication & Pharmacogenomic Model  (Clozapine Subgroup) | |
| --- | --- | --- | --- | --- | --- | --- |
| **Predictors** | **β (SE)** | ***p*** | **β (SE)** | ***p*** | **β (SE)** | ***p*** |
| CPZ-eq Antipsychotic Dose (mg/day) | -0.071 (0.03) | 0.03 | -0.066 (0.03) | 0.047 | -0.134 (0.05) | 0.012 |
| Clozapine current (Yes) | -0.339 (0.07) | 7x10⁻⁷ | -0.339 (0.07) | 1x10⁻⁶ |  |  |
| Anticholinergic (Yes) | -0.276 (0.09) | 0.002 | -0.271 (0.09) | 0.003 | -0.187 (0.15) | 0.203 |
| CYP1A2 Activity Score |  |  | 0.025 (0.03) | 0.433 | 0.066 (0.05) | 0.221 |
| CYP2D6 Activity Score |  |  | -0.037 (0.03) | 0.25 | -0.02 (0.05) | 0.7 |
| CYP3A5 Activity Score |  |  | 0.039 (0.03) | 0.221 | 0.082 (0.05) | 0.117 |
| CYP2C19 Activity Score |  |  | 0.018 (0.03) | 0.57 | 0.005 (0.05) | 0.917 |
| CYP2C9 Activity Score |  |  | -0.025 (0.03) | 0.435 | 0.031 (0.05) | 0.553 |
| NART IQ | 0.475 (0.03) | 3x10⁻⁴² | 0.474 (0.03) | 7x10⁻⁴¹ | 0.454 (0.05) | 5x10⁻¹⁵ |
| Adherent (Yes) | 0.254 (0.14) | 0.073 | 0.26 (0.14) | 0.073 | 0.056 (0.38) | 0.881 |
| Schizophrenia PGS | -0.063 (0.03) | 0.049 | -0.059 (0.03) | 0.068 | -0.097 (0.05) | 0.063 |
| Age | -0.442 (0.03) | 3x10⁻⁴² | 0.438 (0.03) | 8x10⁻³⁶ | -0.481 (0.05) | 1x10⁻¹⁶ |
| Sex (Female) | 0.103 (0.07) | 0.118 | 0.112 (0.07) | 0.094 | 0.053 (0.11) | 0.63 |
| PC1 | 0.074 (0.03) | 0.023 | 0.069 (0.03) | 0.038 | 0.059 (0.05) | 0.276 |
| PC2 | -0.023 (0.03) | 0.446 | -0.022 (0.03) | 0.474 | 0.161 (0.11) | 0.157 |
| PC3 | -0.035 (0.03) | 0.254 | -0.04 (0.03) | 0.199 | -0.509 (0.22) | 0.021 |
| PC4 | 0.027 (0.03) | 0.371 | 0.022 (0.03) | 0.483 | 0.398 (0.21) | 0.061 |
| PC5 | 0.009 (0.03) | 0.778 | 0.004 (0.03) | 0.904 | -0.088 (0.08) | 0.257 |
|  |  |  |  |  |  |  |
| R2 | 0.469 | | 0.470 | | 0.510 | |
| Adjusted R2 | 0.457 | | 0.452 | | 0.465 | |
| N | 558 | | 551 | | 202 | |

Supplementary Table 11. Associations between medication and pharmacogenomic variables with the cognitive ability dimensions in CardiffCOGS. NART IQ is included as a proxy of premorbid intelligence in place of the Intelligence PGS and Educational Attainment PGS used in the analyses presented in the main text. Standardised regression estimates are reported. CPZ-eq = chlorpromazine-equivalent; SE = Standard Error; PGS = Polygenic Score; PC = Genetic Principal Component.

##

## STrengthening the REporting of Genetic Association studies (STREGA) reporting recommendations, extended from STROBE Statement

| **Item** | **Item no** | **STROBE Guideline** | **Extension for Genetic Association Studies (STREGA)** | **Page no** |
| --- | --- | --- | --- | --- |
| **Title and Abstract** | 1 | (a) Indicate the study’s design with a commonly used term in the title or the abstract. |  | Manuscript – title/summary (page 1, 2) |
|  |  | (b) Provide in the abstract an informative and balanced summary of what was done and what was found. |  | Manuscript – summary (page 2) |
| **Introduction** |  |  |  |  |
| *Background rationale* | 2 | Explain the scientific background and rationale for the investigation being reported. |  | Research in context (page 3)  Manuscript – introduction (page 4) |
| *Objectives* | 3 | State specific objectives, including any pre-specified hypotheses | ***State if the study is the first report of a genetic association, a replication effort, or both.*** | Manuscript – introduction (page 5) |
| **Methods** |  |  |  |  |
| *Study design* | 4 | Present key elements of study design early in the paper. |  | Manuscript – introduction and methods (page 4 – 8) |
| *Setting* | 5 | Describe the setting, locations and relevant dates, including periods of recruitment, exposure, follow-up and data collection. |  | Manuscript – methods (page 5) |
| *Participants* | 6 | 1. (a) **Cohort study –** Give the eligibility criteria, and the sources and methods of selection of participants. Describe methods of follow-up.   **Case–control study –** Give the eligibility criteria, and the sources and methods of case ascertainment and control selection. Give the rationale for the choice of cases and controls.  **Cross-sectional study –** Give the eligibility criteria, and the sources and methods of selection of participants. | ***Give information on the criteria and methods for selection of subsets of participants from a larger study, when relevant.*** | Manuscript – methods (page 5)  Figure 2 |
|  |  | **(b) Cohort study** – For matched studies, give matching criteria and number of exposed and unexposed.  **Case–control study –** For matched studies, give matching criteria and the number of controls per case. |  |  |
| *Variables* | 7 | (a) Clearly define all outcomes, exposures, predictors, potential confounders, and effect modifiers. Give diagnostic criteria, if applicable. | ***(b) Clearly define genetic exposures (genetic variants) using a widely –used nomenclature system. Identify variables likely to be associated with population stratification (confounding by ethnic origin).*** | Manuscript – methods (page 5 – 7)  Supplementary materials (page 5) |
| *Data sources measurement* | 8* | (a) For each variable of interest, give sources of data and details of methods of assessment (measurement). Describe comparability of assessment methods if there is more than one group. | ***(b) Describe laboratory methods, including source and storage of DNA, genotyping methods and platforms (including the allele calling algorithm used, and its version), error rates and call rates. State the laboratory /centre where genotyping was done. Describe comparability of laboratory methods if there is more than one group. Specify whether genotypes were assigned using all of the data from the study simultaneously or in smaller batches.*** | Manuscript – methods (page 5 – 7) |
| *Bias* | 9 | (a) Describe any efforts to address potential sources of bias. | ***(b) For quantitative outcome variables, specify if any investigation of potential bias resulting from pharmacotherapy was undertaken. If relevant, describe the nature and magnitude of the potential bias, and explain what approach was used to deal with this.*** | Manuscript – methods (page 6, 7)  Supplementary materials (page 4, 5) |
| *Study size* | 10 | Explain how the study size was arrived at. |  | Manuscript – methods (page 5, 6)  Figure 2 |
| *Quantitative variables* | 11 | Explain how quantitative variables were handled in the analyses. If applicable, describe which groupings were chosen, and why. | ***If applicable, describe how effects of treatment were dealt with.*** | Manuscript – methods (page 5 – 7) |
| *Statistical methods* | 12 | (a) Describe all statistical methods, including those used to control for confounding. | ***State software version used and options (or settings) chosen.*** | Manuscript – methods (page 7, 8) |
|  |  | (b) Describe any methods used to examine subgroups and interactions. |  | Manuscript – methods (page 7, 8)  Supplementary materials (page 5) |
|  |  | (c) Explain how missing data were addressed. |  |  |
|  |  | (d) **Cohort study –** If applicable, explain how loss to follow-up was addressed.  **Case–control study –** If applicable, explain how matching of cases and controls was addressed.  **Cross-sectional study –** If applicable, describe analytical methods taking account of sampling strategy. |  |  |
|  |  | (e) Describe any sensitivity analyses. |  |  |
|  |  |  | ***(f) State whether Hardy- Weinberg equilibrium was considered and, if so, how.*** | Manuscript – methods (page 5) |
|  |  |  | ***(g) Describe any methods used for inferring genotypes or haplotypes.*** | Manuscript – methods (page 6, 7) |
|  |  |  | ***(h) Describe any methods used to assess or address population stratification.*** | Manuscript – methods (page 7, 8) |
|  |  |  | ***(i) Describe any methods used to address multiple comparisons or to control risk of false positive findings.*** | Manuscript – results (page 10) |
|  |  |  | ***(j) Describe any methods used to address and correct for relatedness among subjects.*** | Manuscript – methods (page 5) |
| **Results** |  |  |  |  |
| *Participants* | 13* | (a) Report the numbers of individuals at each stage of the study – e.g. numbers potentially eligible, examined for eligibility, confirmed eligible, included in the study, completing follow-up and analysed. | ***Report numbers of individuals in whom genotyping was attempted and numbers of individuals in whom genotyping was successful.*** | Figure 2 |
|  |  | (b) Give reasons for non-participation at each stage. |  |  |
|  |  | (c) Consider use of a flow diagram. |  |  |
| *Descriptive data* | 14* | (a) Give characteristics of study participants (e.g. demographic, clinical, social) and information on exposures and potential confounders. | ***Consider giving information by genotype.*** | Manuscript – methods (page 6, 8), results (page 9), Table 1, Figure 2  Supplementary materials Supplementary Tables 2 - 3) |
|  |  | (b) Indicate the number of participants with missing data for each variable of interest. |  |  |
|  |  | (c) **Cohort study –** Summarize follow-up time, e.g. average and total amount. |  |  |
| *Outcome data* | 15* | **Cohort study –** Report numbers of outcome events or summary measures over time. | ***Report outcomes***  ***(phenotypes) for each genotype category over time*** | Figure 3  Supplementary materials (Supplementary Table 4) |
|  |  | **Case–control study –** Report numbers in each exposure category, or summary measures of exposure. | ***Report numbers in each genotype category*** |  |
|  |  | **Cross-sectional study –** Report numbers of outcome events or summary measures. | ***Report outcomes (phenotypes) for each genotype category*** |  |
| *Main results* | 16 | (a) Give unadjusted estimates and, if applicable, confounder-adjusted estimates and their precision (e.g. 95% confidence intervals). Make clear which confounders were adjusted for and why they were included. |  | Manuscript – results (page 9 – 11) and Tables 2 – 4.  Supplementary materials (Supplementary Tables 5 – 11) |
|  |  | (b) Report category boundaries when continuous variables were categorized. |  |  |
|  |  | (c) If relevant, consider translating estimates of relative risk into absolute risk for a meaningful time period. |  |  |
|  |  |  | ***(d) Report results of any adjustments for multiple comparisons.*** | Manuscript – results (page 9 – 11) and Tables 2 – 4. |
| *Other analyses* | 17 | (a) Report other analyses done – e.g. analyses of subgroups and interactions, and sensitivity analyses. |  | Manuscript – results (page 9 – 11)  Supplementary materials (page 4, 5) and Supplementary Tables 5 – 11 |
|  |  |  | ***(b) If numerous genetic exposures (genetic variants) were examined, summarize results from all analyses undertaken.*** | Manuscript – Table 3 and Table 4  Supplementary materials (Supplementary Table 6 – 7) |
|  |  |  | ***(c) If detailed results are available elsewhere, state how they can be accessed.*** |  |
| **Discussion** |  |  |  |  |
| *Key results* | 18 | Summarize key results with reference to study objectives. |  | Manuscript – discussion (page 11) |
| *Limitations* | 19 | Discuss limitations of the study, taking into account sources of potential bias or imprecision. Discuss both direction and magnitude of any potential bias. |  | Manuscript – discussion (page 11 – 15) |
| *Interpretation* | 20 | Give a cautious overall interpretation of results considering objectives, limitations, multiplicity of analyses, results from similar studies, and other relevant evidence. |  | Manuscript – discussion (page 11 – 15) |
| *Generalizability* | 21 | Discuss the generalizability (external validity) of the study results. |  | Manuscript – discussion (page 11 – 15) |
| **Other information** |  |  |  |  |
| *Funding* | 22 | Give the source of funding and the role of the funders for the present study and, if applicable, for the original study on which the present article is based. |  | Manuscript – abstract (page 2)  Acknowledgements (page 17, 18) |

## Supplementary References

1 Nuechterlein KH, Green MF, Kern RS, *et al.* The MATRICS Consensus Cognitive Battery, Part 1: Test Selection, Reliability, and Validity. *AJP* 2008; **165**: 203–13.

2 Tracy JI, McGrory AC, Josiassen RC, Monaco CA. A comparison of reading and demographic-based estimates of premorbid intelligence in schizophrenia. *Schizophrenia Research* 1996; **22**: 103–9.

3 Food and Drug Administration. Drug Development and Drug Interactions: Table of Substrates, Inhibitors and Inducers. 2023; published online April 12. https://www. fda.gov/drugs/drug-interactions-labeling/drug-development-and-druginteractions-table-substrates-inhibitors-and-inducers (accessed Feb 9, 2024).

4 Klomp SD, Manson ML, Guchelaar H-J, Swen JJ. Phenoconversion of Cytochrome P450 Metabolism: A Systematic Review. *Journal of Clinical Medicine* 2020; **9**: 2890.

5 Mostafa S, Polasek TM, Sheffield LJ, Huppert D, Kirkpatrick CMJ. Quantifying the Impact of Phenoconversion on Medications With Actionable Pharmacogenomic Guideline Recommendations in an Acute Aged Persons Mental Health Setting. *Front Psychiatry* 2021; **12**: 724170.

6 Hukkanen J, Jacob P, Peng M, Dempsey D, Benowitz NL. Effect of nicotine on cytochrome P450 1A2 activity. *British Journal of Clinical Pharmacology* 2011; **72**: 836–8.

7 Coustals N, Martelli C, Brunet-Lecomte M, Petillion A, Romeo B, Benyamina A. Chronic smoking and cognition in patients with schizophrenia: A meta-analysis. *Schizophrenia Research* 2020; **222**: 113–21.

8 Lesche D, Mostafa S, Everall I, Pantelis C, Bousman CA. Impact of CYP1A2, CYP2C19, and CYP2D6 genotype- and phenoconversion-predicted enzyme activity on clozapine exposure and symptom severity. *The Pharmacogenomics Journal* 2020; **20**: 192–201.

9 Saiz-Rodríguez M, Ochoa D, Belmonte C, *et al.* Polymorphisms in CYP1A2, CYP2C9 and ABCB1 affect agomelatine pharmacokinetics. *Journal of Psychopharmacology* 2019; **33**: 522–31.

10 Lee S, Shin J-Y, Kwon N-J, Kim C, Seo J-S. ClinPharmSeq: A targeted sequencing panel for clinical pharmacogenetics implementation. *PLOS ONE* 2022; **17**: 1–19.

11 Whirl-Carrillo M, Huddart R, Gong L, *et al.* An Evidence-Based Framework for Evaluating Pharmacogenomics Knowledge for Personalized Medicine. *Clinical Pharmacology & Therapeutics* 2021; **110**: 563–72.
